# Development of a viability digital PCR protocol for the selective detection and quantification of live *Erwinia amylovora* cells in cankers

**DOI:** 10.1038/s41598-019-47976-x

**Published:** 2019-08-08

**Authors:** Ricardo D. Santander, Christopher L. Meredith, Srđan G. Aćimović

**Affiliations:** Plant Pathology and Plant-Microbe Biology Section, Cornell University, Hudson Valley Research Laboratory, Highland, NY USA

**Keywords:** Microbiology techniques, Bacterial techniques and applications

## Abstract

Fire blight is a devastating disease of apple and pear caused by the bacterium *Erwinia amylovora*. One of its main symptoms is canker formation on perennial tissues which may lead to the death of limbs and/or the entire tree. *E. amylovora* overwinters in cankers which play an important role in initiating fire blight epidemics. However, knowledge of pathogen biology in cankers is scarce, in part due to limitations of classical microbiology methods and the inability of most molecular techniques to distinguish live from dead cells. In this work, a viability digital PCR (v-dPCR) protocol using propidium monoazide (PMA) was developed, allowing for the first time the selective detection and absolute quantification of *E. amylovora* live cells in apple and pear cankers collected in two time periods. Some key factors affecting the v-dPCR performance were the maceration buffer composition, the target DNA amplicon length, the thermal cycle number and the use of sodium dodecyl sulfate or PMA enhancer for Gram-negative bacteria to improve the effect of PMA. In the future, this methodology could shed light on *E. amylovora* population dynamics in cankers and provide clues on the effect of management practices, host cultivar, host water/nutritional status, etc., on bacterial survival.

## Introduction

*Erwinia amylovora* is the etiological agent of fire blight of rosaceous plants, a devastating plant disease affecting economically important pome fruit crops like apple (*Malus pumila* Mill.) and pear (*Pyrus communis* L.), as well as ornamental and wild species^1,2^. Fire blight is a systemic disease attacking almost every plant organ, causing necrosis and characteristic exudates in actively growing tissues, and formation of cankers in the perennial ones, mainly on branches, the trunk, and/or the rootstock. *E. amylovora* overwinters in cankers until spring, when favorable environmental conditions break the host’s winter dormancy. With the host’s growth renewal, the pathogen cells multiply and emerge on the surface of some cankers, serving as the primary inoculum source for new disease outbreaks^1,3^.

While a role of other reservoirs in fire blight epidemics has been discussed^4–6^, cankers are widely considered one of the main sources of *E. amylovora* cells for the spread of the disease. However, knowledge of *E. amylovora* population dynamics in cankers through time and the impact of environmental and/or host-specific factors on *E. amylovora* survival in cankers is scarce, partially due to limitations of classical microbiology detection methods employed in plant disease diagnostics. Most attempts to determine the presence of *E. amylovora* in cankers have focused on the isolation on culture media and/or classical PCR^7–11^. Culture-dependent methods can underestimate the number of viable bacteria due to the impaired growth of stressed cells, growth inhibition by competitive microbiota, and/or the existence of pathogen cell populations in the viable but nonculturable (VBNC) state, which involves the inability of live bacteria to form colonies on solid media^12^. On the other hand, classical PCR detection neither discriminates between the live and dead *E. amylovora* cells nor allows their quantification. Improvement of molecular methods for pathogen quantification and/or selective detection of viable cells have been two important research topics in the last two decades^13–18^.

Digital PCR (dPCR) is a technology gaining importance in the field of plant pathology^19–22^. This technique builds on traditional PCR amplification and fluorescent probe–based detection methods such as quantitative PCR (qPCR), while enabling the absolute quantification of nucleic acids without requiring standard curves. This makes interlaboratory comparison of quantification data easier and less laborious. The main feature distinguishing dPCR from other PCR variants is the partition of samples into thousands of independent PCR sub-reactions, so that each partition receives either one or no target DNA sequences. End-point PCRs occur in parallel in each individual partition. The positive and negative amplification reactions are detected and quantified by means of fluorescence and the final concentration of target DNA copies in the sample is determined by Poisson distribution statistics^23,24^.

Similar to qPCR, dPCR allows the detection and quantification of specific DNA targets, but it is unable to determine if the amplified genetic material comes from live or dead cells. Many works have attempted to use the viability PCR dye propidium monoazide (PMA) for selective amplification of live bacterial DNA^25–28^. PMA is a DNA intercalating agent able to penetrate only compromised dead cell membranes. After photo-activation, PMA binds covalently to DNA and inhibits amplification by polymerases. Hence, only DNA originating from live cells can be detected by PCR^29^. However, despite the promising uses of PMA for molecular detection of live cells, several studies have highlighted major drawbacks of the technique leading to detection of false positives^30^.

In this study we developed a viability dPCR (v-dPCR) protocol for *E. amylovora* combining the chip-based QuantStudio 3D (QS3D) dPCR system and PMA. After optimization, v-dPCR allowed selective detection and absolute quantification of live *E. amylovora* cells in natural apple and pear cankers. This newly developed methodology will allow investigation of unknown aspects of *E. amylovora* biology including host interactions, pathogen population dynamics during canker formation and maturation, detection of nonculturable cells in plant samples, and assessment of the effect of environmental and/or host-related factors on *E. amylovora* survival in plant tissues.

## Results and Discussion

### Direct transfer of a qPCR protocol to the QS3D dPCR system

A known qPCR protocol for *E. amylovora*^31^ was transferred to the QS3D dPCR platform, using the same primer and probe concentrations and thermal cycling conditions as a first step to test the QS3D dPCR technology.

Analysis of no-template negative controls and DNA samples from *E. amylovora-*free plant material extracts resulted in detection of a background fluorescence signal easily distinguished from that corresponding to the positive amplification of target DNA (i.e. positive fluorescence calls) (Supplementary Fig. S1). However, a variable number of positive calls (0–5) was detected in some of the negative controls prepared with *E. amylovora-*free apple and pear plant macerates, with fluorescence intensity values similar to those in positive controls (Supplementary Fig. S2). These calls might be produced by cross-reaction with DNA of the host or common microorganisms present in samples, as reported previously^19^. Based on these results, only samples containing more than 5 positive calls per chip were considered positive for *E. amylovora* detection in further assays.

**Figure 1:**
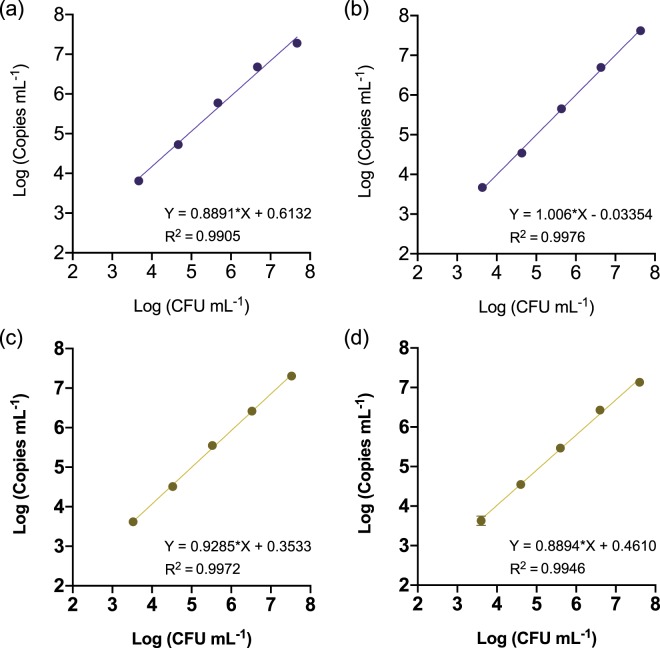
Correlation between the Log-transformed values of plate counts and QS3D dPCR absolute quantification values. Serial tenfold dilutions of *E. amylovora* ATCC 49946 (**a**,**b**) and CFBP 1430 (**c**,**d**) were performed in apple (**a**,**c**) and pear (**b**,**d**) branch macerates prepared in AMB, followed by DNA extraction and dPCR using a protocol transferred from Pirc *et al*.^31^. Dots and error bars are average values of three biological repeats of the experiment ± SD. Absent error bars indicate SD values smaller than the represented symbols.

Correlation assays showed a linear relationship with a high correlation index (R^2^ > 0.99) between colony numbers and target DNA copy counts by dPCR in the range of 10^3^ to 10^7^ CFU mL^−1^ (Fig. 1). Pathogen detection in samples containing bacterial concentrations below 10^3^ CFU mL^−1^ or above 10^7^ CFU mL^−1^ led to inconsistent results or inaccurate quantification values due to chip saturation, respectively (Supplementary Tables S1 and S2).

The results in this section were similar for both *E. amylovora* strains (ATCC 49946 and CFBP 1430) and hosts (apple and pear), suggesting the QS3D dPCR system as a robust technology potentially applicable for analysis of host plant material contaminated with different strains of the pathogen.

### Live-cell numbers are overestimated by v-dPCR using standard PMA treatments and the dPCR protocol transferred from a qPCR assay

**Figure 2:**
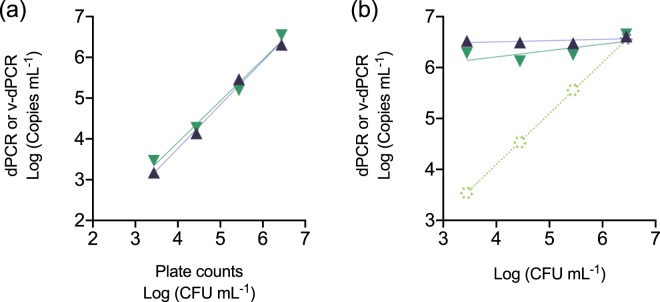
Correlation between plate counts and v-dPCR values in the presence of dead cells. Apple macerates prepared in AMB were inoculated with *E. amylovora* live cells ranging from 10^3^ to 10^6^ CFU mL^−1^ (control) (**a**), or the same range of live cell concentrations, but with a constant background of 10^6^ dead cell mL^−1^ (**b**). Each dilution was subjected to dPCR to quantify total target DNA copies mL^−1^ (purple), and v-dPCR, to quantify the copies mL^−1^ coming from live cells (green), using primers and probe of Pirc *et al*.^31^. The linear regression equations and coefficients of correlation corresponding to dPCR and v-dPCR values in (**a**) were Y = 1.070*X–0.5165 (R^2^ = 0.9925) and Y = 1.014*X–0.1432 (R^2^ = 0.9825), respectively. Dashed lines and symbols in (**b**) represent the expected live cell concentration values (pale green), calculated based on plate counts of the live cell stock used to prepare samples. The slopes of the regression lines corresponding to dPCR and v-dPCR values in (**b**) did not statistically differ from 0 (p > 0.05). In both graphs, each value is the mean of three independent repeats. Absent error bars indicate SD values smaller than the represented symbols.

To evaluate the use of PMA for selective detection of live cells, apple plant macerates were artificially inoculated either with increasing live *E. amylovora* cell concentrations, or with the same live cell concentrations in a background of dead *E. amylovora* cells. Aliquots of the same samples were analyzed by dPCR and v-dPCR to calculate total and viable cell numbers, respectively. A good correlation between the inoculated CFU mL^−1^ and the total (R^2^ > 0.99) and live (R^2^ > 0.98) cell concentrations was observed in samples inoculated with the increasing live cell concentrations (Fig. 2a). However, the analysis of mixed live/dead samples revealed the failure of v-dPCR to distinguish live from dead cells, with live cell concentration values being close to those of total cell counts (i.e. most of the dead cells in mixed samples were quantified as live ones by v-dPCR) (Fig. 2b).

The overestimation of live cells in PMA-treated samples has also been documented in previous works^32,33^. Factors conditioning PCR inhibition by PMA are the dye capacity to penetrate dead cell membranes and a concentration ensuring its binding to all the target DNA copies in the sample. Accordingly, insufficient PMA concentrations and/or the use of killing methods causing insufficient bacterial membrane damage might lead to the detection and quantification of dead cells as live ones.

**Figure 3:**
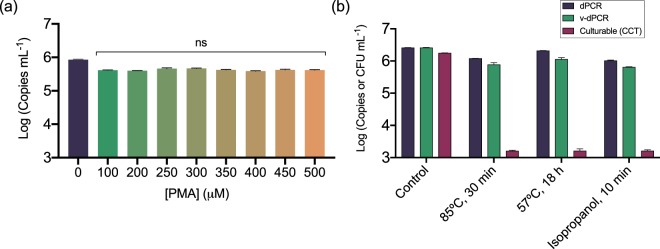
Effect of PMA concentration and the *E. amylovora* killing method on the exclusion of false-positive signals by v-dPCR. Apple branch macerates prepared in AMB were inoculated with 10^6^ *E. amylovora* heat-killed cells mL^−1^ (85 °C for 30 min), and treated with PMA concentrations ranging from 0–500 μM before DNA extraction and dPCR (**a**). *E. amylovora* live cells (10^3^ CFU mL^−1^) were mixed with dead cells (10^6^ cell mL^−1^) obtained either by heating at 85 °C for 30 min, heating at 57 °C for 18 h, or exposure to 70% isopropanol for 10 min. Samples were then analyzed by dPCR, v-dPCR (treatment with 100 μM PMA) and plate counts (**b**). D-PCRs were performed using the same primers and probe as Pirc *et al*.^31^. Represented data are mean values of experiments performed in triplicate. Error bars show the SD.

The PMA concentration and photo-activation conditions used in this work were based on other works analyzing plant material and/or more complex samples^26,27^. The use of high PMA concentrations, up to 500 μM, did not have an apparent effect on false-positive signal suppression in samples containing only heat-killed *E. amylovora* cells (Fig. 3a). This indicated that almost all the dead cells were detected and quantified as live cells regardless of the PMA concentration. Similarly, the PMA treatment of mixtures of *E. amylovora* live and dead cells obtained by different killing methods (85 °C, 30 min; 57 °C, 18 h; 70% isopropanol, 10 min) revealed no apparent effects of the killing method on the suppression of false-positive signals coming from the dead cells (Fig. 3b). Hence, based on these results, the overestimation of *E. amylovora* live cells by v-dPCR did not seem to be particularly associated with an insufficient PMA concentration or an inappropriate killing method.

### Improvement of v-dPCR false-positive suppression by targeting larger DNA sequences

Another important parameter conditioning the potential of v-dPCR to discriminate between live and dead cells is the length of the target DNA determined by primers^30,34^. Similar to propidium iodide, PMA does not have a preference for specific nucleotides or DNA sequences, so the dye binding to DNA follows a random distribution. The employed PMA concentrations for viability-PCR are far from saturation^34^. Therefore, the probability of PMA to bind the target DNA and inhibit PCR amplification increases with larger amplicon sizes. Likewise, greater numbers of thermal cycles might increase the probability to amplify target DNA from a dead cell and/or to detect the fluorescence generated by false positive reactions.

**Figure 4:**
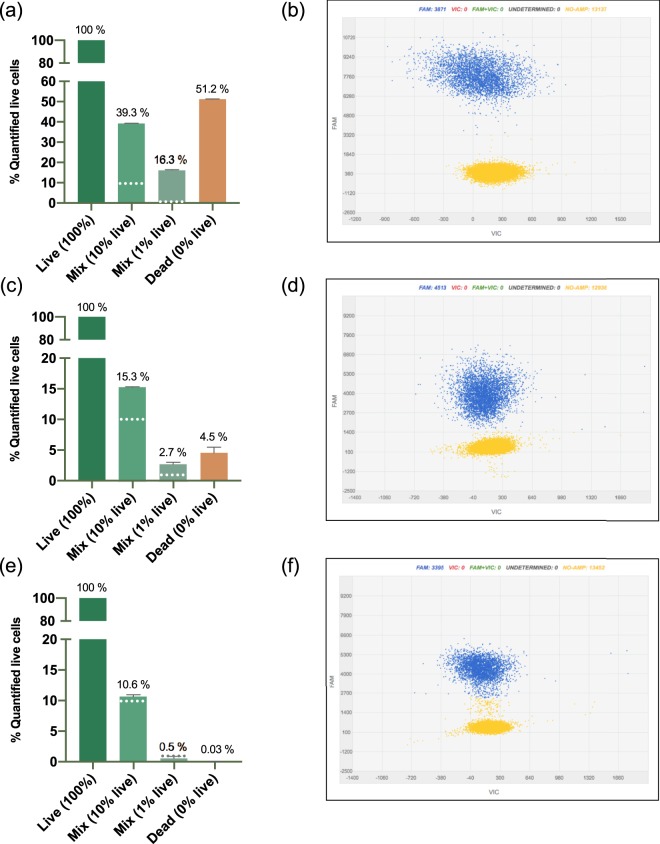
Effect of the thermal cycle number and the amplicon size on the reduction of false-positive signals by v-dPCR. Apple branch macerates prepared in AMB were inoculated with either *E. amylovora* at 7 × 10^6^ CFU mL^−1^ (100% live cells), 7 × 10^6^ dead cells mL^−1^ (0% live cells), or live/dead cell mixtures containing a 10% and a 1% of live cells and a 90% and a 99% of dead cells, respectively. After PMA treatment and DNA extraction, dPCR was conducted using: *i)* same primers and probe as Pirc *et al*.^31^, with 40 amplification cycles (**a**,**b**); *ii)*, reducing the number of thermal cycles to 30 (**c**,**d**); *iii)* using a primers targeting a 966 bp DNA sequence, and 40 amplification cycles (**e**,**f**). Each column shows mean values of two independent experiments. Error bars are the SD. Dashed horizontal lines indicate the theoretical percentage of live cells in samples containing live/dead cell mixtures (10% and 1%). Representative dPCR 2D scatter plots showing fluorescence values of the FAM labelled probe in wells with positive calls (blue) and fluorescent values corresponding to negative amplifications (yellow) (**b**,**d**,**f**).

The effects of a drastic reduction of thermal cycles from 40 to 30 and the use of a large amplicon size of 966 bp versus 74 bp were tested to determine the next steps for v-dPCR optimization (Fig. 4). Compared to the original protocol (74 bp amplicon; 40 thermal cycles) (Fig. 4a,b), both the reduction of thermal cycles (Fig. 4c,d) and the increase of the amplicon size (Fig. 4e,f) had a positive effect on the suppression of false positive signals by v-dPCR in samples containing dead *E. amylovora* cells, either mixed or not with live cells. However, the reduction of cycle numbers had a strong impact on fluorescence intensity values, which on average, became closer to the ones  corresponding to no-template reactions (Fig. 4d).

Based on these results, new primers targeting DNA sequences with lengths ranging between 74 bp and 966 bp (Table 1) were designed and their effect on false-positive signal suppression analyzed. The new primers were specific against a collection of 30 *E. amylovora* strains, including reference strains and natural isolates. They provided negative amplification results with 16 other bacterial species, including bacterial phytopathogens of the genera *Dickeya, Pectobacterium, Pantoea* and *Pseudomonas*, as well as unidentified saprophytic bacteria usually isolated together with *E. amylovora* from apple cankers on CCT medium (Supplementary Table S3).

**Table 1:** Primer and probe combinations used in this study.

Primer/Probe	Sequence (5′ –> 3′)	Reference	Amplicon size in combination with Ams189R
**Primers**			
Ams189R^†^	GGG TAT TTG CGC TAA TTT TAT TCG	Pirc *et al*., 2009	
Ams116F	TCC CAC ATA CTG TGA ATC ATC CA	Pirc *et al*., 2009	74 bp
Ams03KbF	ATA TCC TTG TTG ATA AAC AGG TGC G	This study	325 bp
Ams06KbF^†^	AAT TGG TTC CGC TAT AAC TTG CAG	This study	627 bp
Ams09KbF	TAA TAG ACT GAG CTG AAA GTA TCG TGT	This study	966 bp
**Probe**			
Ams141T	FAM-CCA GAA TCT GGC CCG CGT ATA CCG-TAMRA	Pirc *et al*., 2009	

^†^Primer combination used in the final optimized protocol for dPCR and v-dPCR.

**Figure 5:**
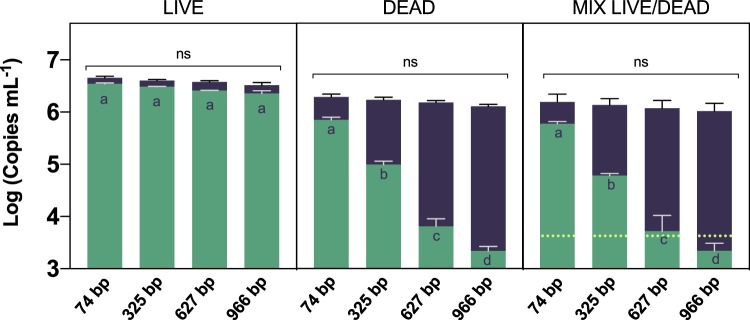
Effect of the amplicon length on the suppression of false positive signals by v-dPCR. Apple branch macerates prepared in AMB were inoculated with *E. amylovora* live (10^6^ CFU mL^−1^), dead (10^6^ dead cells mL^−1^) or a defined ratio of live (10^3^ CFU mL^−1^) and dead cells (10^6^ dead cell mL^−1^). Samples were sub-aliquoted for total (purple) and live (green) cell quantification by dPCR and v-dPCR, respectively, using primer combinations targeting a 74, 325, 627 and 966 bp DNA sequence. Data are mean values of three biological repeats of the same experiment. Error bars show the SD. Different letters denote statistically significant differences between live cell counts of samples treated with PMA. Non-statistically significant (ns) differences were detected between dPCR total cell counts regardless of the amplicon length.

The amplicon size had no effect on dPCR total cell quantification, regardless of the cell viability status (*p* > 0.05) (Fig. 5). Similarly, the increase of the amplicon length did not affect the v-dPCR outcome during the analysis of samples composed of only live cells (*p* > 0.05), but was linked to a significant reduction of false-positive signals in samples containing dead cells (*p* < 0.0001) (Fig. 5).

The v-dPCR analysis of defined mixtures of live and dead cells using the primer combination targeting a 966 bp DNA sequence underestimated the number of live cells with respect to the CFUs inoculated per sample (*p* < 0.05). Cell number underestimation associated with large amplicon lengths for qPCR is usually attributed to accumulation of DNA damage that, similarly to PMA, inhibits or reduces the efficiency of amplification by the DNA polymerase^34^. Based on these results, the 966 bp amplicon size was discarded for further assays. The v-dPCR analysis of the same samples with the primer combination targeting a 627 bp DNA sequence provided viability values slightly above, but non-significantly different to, the inoculated CFUs (*p* < 0.05). However, the use of smaller amplicon lengths, led to a clear overestimation of viable cell counts with respect to the inoculated CFUs (*p* < 0.05).

### Sample processing in diluted AMB and the addition of a membrane destabilizer during PMA treatments enhance false-positive suppression by v-dPCR

The sample matrix may interfere with v-dPCR by complexing or reacting with PMA molecules, thus impeding the ability of this viability dye to penetrate dead cell membranes and/or bind to DNA, as well as by hampering PMA photo-activation. In other studies, PMA concentrations and photo-activation protocols similar to the ones used in this work have been used to analyze plant material^27,35,36^ and more complex samples with different degrees of turbidity and particle size^26^.

Sample processing is usually conducted in water or a buffered solution. We used anti-oxidant maceration buffer (AMB)^37^ as recommended by European and American plant protection organizations^38,39^ for *E. amylovora* diagnostics. This solution buffers pH around neutral and contains a variety of antioxidants which improve the isolation of stressed *E. amylovora* cells on culture media and remove PCR inhibitors during DNA purification^37–39^. However, some of the AMB components might also interact with PMA, DNA, cell membranes or all of them.

Several works have reported an additional improvement of viability qPCR by complementing the viability dye treatment with a membrane destabilizer^40,41^. Similarly, a commercial product, PMA Enhancer for Gram-negative bacteria, improving PMA affinity to dead-cell DNA can be combined with PMA to improve the selective detection of live cells.

**Figure 6:**
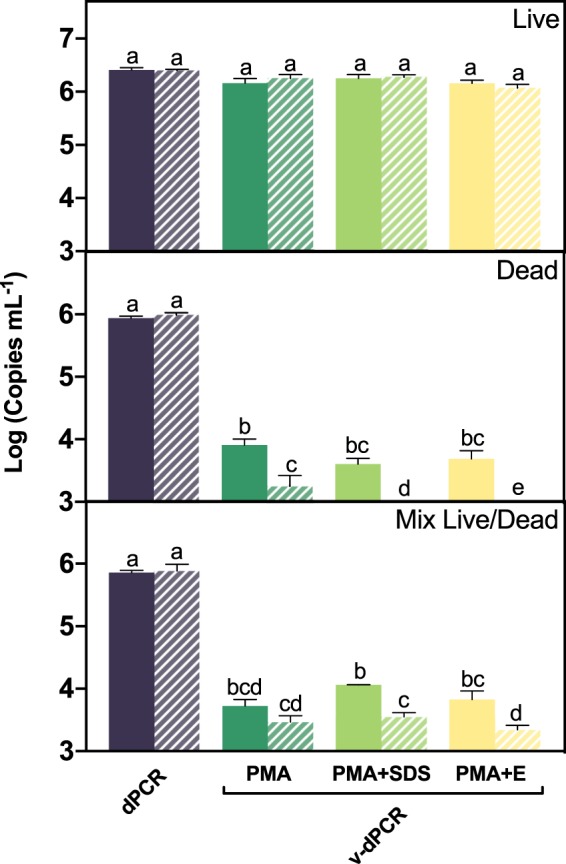
Effect of the maceration buffer, SDS and PMA Enhancer for Gram negative bacteria on the suppression of *E. amylovora* dead cell signals by v-dPCR. Apple branch macerates prepared either with AMB (solid columns) or 0.1xAMB (dashed columns) were inoculated with *E. amylovora* live (10^6^ CFU mL^−1^), dead (10^6^ dead cells mL^−1^) or a mixture of live (10^3^ CFU mL^−1^) and dead cells (10^6^ dead cell mL^−1^). Total cell numbers were quantified by dPCR. The effect of SDS and PMA Enhancer on viable cell quantification was assessed comparing v-dPCR viable cell counts of samples (i) only treated with PMA (100 μM); (ii) PMA plus 0.0025% (w/v) SDS (PMA + SDS); (iii) PMA plus PMA Enhancer (PMA + E). For both, total and viable cell quantification, primers targeting a 627 bp DNA sequence were used. Columns represent mean values of three independent experiments in duplicate. Error bars show the SD. Different letters denote statistically significant differences.

We determined the effect of the maceration buffer and the addition of the membrane destabilizer sodium dodecyl sulfate (SDS) or PMA Enhancer during PMA treatments. Suspensions of *E. amylovora* live, dead, and a mixture of live and dead cells were prepared in plant macerates obtained in either AMB or tenfold diluted AMB (0.1xAMB). Additionally, v-dPCR was performed using either PMA, PMA plus 0.0025% SDS (w/v) (PMA + SDS), or PMA plus PMA Enhancer (PMA + E) (Fig. 6). The SDS assay concentration was chosen based on a minimum inhibitory concentration assay (Supplementary Fig. S3). The dPCR reactions were performed using the primer combination Ams06KbF/Ams189R (627 bp amplicon size), which in a previous experiment (Fig. 5) significantly reduced false positive detection providing v-dPCR quantification values similar to the inoculated CFU mL^−1^.

Total cell quantification by dPCR was similar (*p* > 0.05) in samples processed in AMB and in 0.1xAMB, regardless of the type of sample analyzed (live, dead, live/dead mix) (Fig. 6). Viable cell counts (v-dPCR) in samples composed of only live cells were also similar (*p* > 0.05) regardless of the maceration buffer or the PMA treatment employed to inhibit dead-cell DNA amplification (Fig. 6). However, the two-way ANOVA analysis revealed an effect of both the maceration buffer (*p* < 0.001) and the PMA treatment (*p* < 0.05) in samples containing dead cells, alone and mixed with live cells (Fig. 6). In samples containing only heat-killed cells, a total exclusion of dead-cell signals was achieved by using 0.1xAMB and PMA treatments combined with SDS or PMA Enhancer (Fig. 6). In samples containing mixed live/dead cells, the effect of the maceration buffer concentration was only significant in samples treated with PMA + SDS or PMA + E (p < 0.01), but not in the samples treated only with PMA (*p* > 0.05).

The same samples were analyzed using the primer pair targeting the 325 bp DNA sequence. However, the false-positive signals were consistently detected regardless of the maceration buffer concentration and the type of PMA treatment, confirming that a larger amplicon size is required to efficiently remove signals coming from dead cells (Supplementary Fig. S4).

These results show that, while AMB does not seem to have a detrimental effect on total cell quantification by dPCR, one or more AMB components are probably involved in the failure of PMA treatments to suppress false-positive signals. This hypothesis is also supported by the fact that extremely high PMA concentrations of up to 500 μM in samples processed with AMB did not inhibit the DNA polymerase (Fig. 3a), as reported by other authors using lower concentrations of PMA^33,42^. The most concentrated AMB component is polyvinylpyrrolidone (PVP) (2% w/v), a polymer used in DNA extraction protocols due to its ability to remove PCR inhibitors present in plant material. However, PVP also interacts with DNA^43^, with the PVP-DNA complex possessing less negative charge density and being more hydrophobic than DNA alone^43^. Although PVP interactions with DNA do not affect PCR amplification, it could negatively impact the PMA capacity to bind DNA. Further studies are required to determine which of the AMB compound/s negatively affect v-dPCR and by what mechanism.

### *In vitro* performance of the dPCR and the optimized v-dPCR in samples with increasing live cell concentrations

The previous assay revealed the conditions allowing a complete dead-cell signal suppression by v-dPCR: (i) sample processing in 0.1xAMB, (ii) treatment with PMA + E (or SDS), (iii) and dPCR amplification using the primers Ams06KbF/Ams189R (Fig. 6).

**Figure 7:**
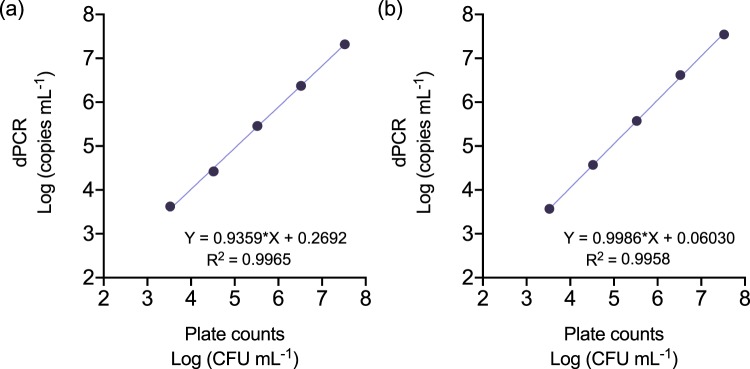
Correlation between the Log-transformed values of plate counts and QS3D dPCR absolute quantification values, using primers Ams06KbF and Ams189R. Serial tenfold dilutions of *E. amylovora* were performed in apple (**a**) and pear (**b**) branch macerates prepared in 0.1xAMB, and subjected to DNA extraction and dPCR. Each dot represents the average value of two biological repeats performed in triplicate. Absent error bars indicate SD values smaller than the represented symbols.

**Figure 8:**
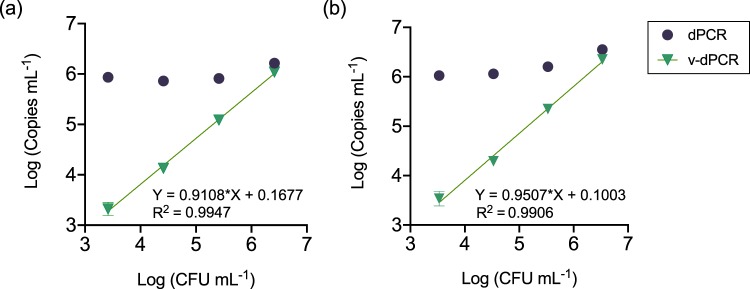
Correlation between plate counts and v-dPCR values in the presence of dead cells, using improved methods for plant material processing and dPCR. Apple and pear branch macerates prepared in 0.1xAMB were inoculated with increasing live cell concentrations (from 10^3^ to 10^6^ CFU mL^−1^) in a constant background of 10^6^ dead cell mL^−1^. Total cell counts were performed by dPCR. For viable cell counts, samples were treated with PMA plus 1x PMA Enhancer before dPCR. In all cases, dPCR was performed using primers targeting a 627 bp DNA sequence. Each dot corresponds to mean values of two independent experiments performed in triplicate. Error bars show the SD. Absent error bars indicate SD values smaller than the represented symbols. Solid lines represent the linear regression best fit through all the v-dPCR dataset points. The corresponding linear regression equations and coefficients of correlation (R^2^) are also indicated.

Before testing these conditions with natural samples, we first characterized the dPCR performance with primers Ams06KbF/Ams189R, by analyzing increasing *E. amylovora* live cell concentrations in apple and pear plant macerates prepared in 0.1xAMB (Fig. 7). Afterwards, we also tested the efficiency of the new v-dPCR conditions (sample processing with 0.1xAMB, treatment with PMA + E, dPCR with primers Ams06KbF/Ams189R) in quantifying increasing *E. amylovora* live cell concentrations in the presence of dead cells (Fig. 8).

The dPCR quantification range using primers Ams06KbF and Ams189R (Fig. 7; Table 2) was 10^3^ to 10^7^ CFU mL^−1^, similar to the one with primers Ams116F/Ams189R (74 bp amplicon)^31^ in this work (Fig. 1) and in a previous one using a different dPCR platform^19^. Other quantitative techniques provide wider ranges of detection and quantification. However, the lack of requirement of calibration controls makes dPCR a good option to simplify the analysis of natural samples and allows their comparability^23^. The mean coefficients of variation (%CV) of two independent experiments calculated for each of the assayed bacterial concentrations (from 10^3^ to 10^7^ CFU mL^−1^), ranged from 1.23 to 23.36% in apple, and 1.26–24.78% in pear. The highest %CV were observed in samples containing 10^3^ CFU mL^−1^, and the lowest ones in samples containing 10^6^ CFU mL^−1^. Bacterial concentrations below 10^3^ CFU mL^−1^ and higher than 10^7^ CFU mL^−1^ led to unsuccessful detection of *E. amylovora* cells in most of the replicates and to chip saturation, respectively, similar to preliminary experiments using the primers designed by Pirc *et al*.^31^ (Table 2). Therefore, the limit of detection and quantification for our newly developed dPCR protocol had the same value regardless of the analyzed plant material, ca. 10^3^ CFU mL^−1^ (i.e. the pathogen was detected in all the analyzed replicates, with a %CV below 25%).

**Table 2:** Characterization of the QS3D dPCR performance using the primer combination Ams06KbF/Ams189R, targeting a 627 bp DNA sequence.

Host	Log CFU mL^−1^ ^b^	Log Copies mL^−1^ (±SD)^c,d^	Copies rxn^−1^ (±SD)^d,e^	# Qualified by QT (± SD)^d,f^	# Pos (±SD)^d,g^
**Apple cv. Honeycrisp** ^a^	7.33	7.32 ± 0.06	4.406 ± 0.623	17353 ± 279	17057 ± 190
	6.33	6.37 ± 0.01	0.494 ± 0.014	17537 ± 195	6824 ± 183
	5.33	5.46 ± 0.06	0.061 ± 0.009	17205 ± 812	1021 ± 183
	4.33	4.42 ± 0.08	0.005 ± 0.001	17656 ± 381	93 ± 20
	3.33	3.62 ± 0.10	0.001 ± 0.000	17388 ± 339	14 ± 5
**Pear cv. Bosc** ^a^	7.33	7.54 ± 0.10	4.328 ± 0.988	17725 ± 630	17351 ± 423
	6.33	6.62 ± 0.11	0.518 ± 0.126	15655 ± 602	7019 ± 1101
	5.33	5.57 ± 0.10	0.047 ± 0.010	17752 ± 829	802 ± 139
	4.33	4.57 ± 0.08	0.005 ± 0.001	17490 ± 393	80 ± 16
	3.33	3.62 ± 0.09	0.000 ± 0.000	17707 ± 705	8 ± 2

^a^*E. amylovora-*free plant material was processed in a plastic bag containing 0.1xAMB in a ratio 1:50 (w/v), by hammering.

^b^Data shown in this column correspond to average bacterial concentrations of two different *E. amylovora* stocks, employed to inoculate samples in two independent assays performed in triplicate. Values in each dilution were calculated based on the stock concentrations. Hence, the SD in all cases was the same, ±0.24.

^c^Log Copies mL^−1^ = Log [copies μL^−1^_rxn_ × 1/D_rxn tube_  × 1/E_DNA Extr_  × 1/Vol_Pl.Macerates_]; i.e. Log (copies μL^−1^_rxn_ × 1.79 × 10^3^) for Apple, and Log (copies μL^−1^_rxn_ × 4.81 × 10^3^) for pear.

^d^Average values of two independent experiments performed in triplicate.

^e^Quantity of target DNA copies, expressed in copies per reaction well.

^f^Total number of reaction wells in the chip fitting the selected quality threshold.

^g^Number of positive calls for FAM dye in the chip, as determined from the Review Calls scatter plot. Based on the no-template control and plant material negative control analysis, chips with 5 or less than 5 positive calls were considered negative for *E. amylovora* detection, and the provided dPCR values were not considered for quantification.

Apple and pear plant macerates containing increasing live *E. amylovora* cell concentrations with a background of dead cells were used to determine the ability of the optimized sample processing protocol and v-dPCR conditions to suppress false-positive signals during live cell quantification (Fig. 8). Regardless of the analyzed plant material (apple or pear), dPCR analysis of samples provided total cell numbers coinciding with the inoculated bacterial concentrations, around 10^6^ mixed live/dead cells mL^−1^. Similarly, v-dPCR live cell values correlated well (R^2^ > 0.99) with the inoculated CFU mL^−1^ despite the assayed ratio of live:dead cells ranging from 1:1 in samples containing 10^6^ live cells mL^−1^ and 10^6^ dead cells mL^−1^, to 1:999 in samples containing 10^3^ live cells mL^−1^ plus 10^6^ dead cells mL^−1^ (Fig. 8). Live:dead cell ratios higher than 1:999 were also tested, but led to an overestimation of live cells, similar to that reported by other authors^32,33^. Hence, the method seems suitable for the analysis of samples containing relevant concentrations of live bacterial cells without an excessive load of dead bacteria.

### Absolute quantification of *E. amylovora* live cells in natural cankers

Although many works have addressed the characterization of cankers^8,44–46^, accurate estimations of live *E. amylovora* populations in these structures are limited and usually based on culture dependent methods. We tested the sample processing with 0.1xAMB, primers targeting a 625 bp DNA sequence, and different PMA treatments for v-dPCR to analyze apple and pear cankers originating from fire blight infections occurring in early spring and harvested in mid-summer and mid-winter. The assayed PMA treatments consisted of PMA, PMA + SDS, PMA + E, and equivalent treatments using PMAxx, which is an improved commercial version of PMA. Total and culturable cell numbers were also quantified by dPCR (using the same primers) and plate counts on SNAN and CCT media, respectively.

**Figure 9:**
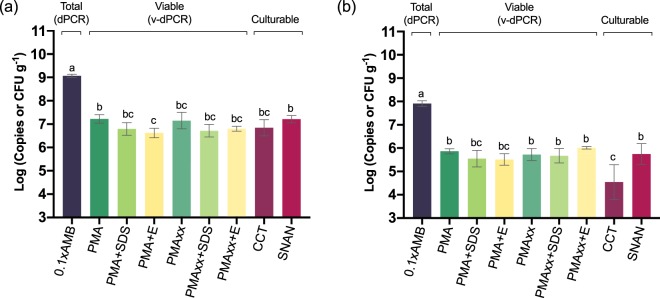
*E. amylovora* total, live and culturable cell populations in fire blight cankers on apple (cv. Honeycrisp). Fire blight infections started in early-spring of 2016, and cankers were collected in mid-summer (July 2016) (**a**) and mid-winter (January 2017) (**b**). Samples were homogenized with 0.1xAMB. Total cell counts were performed by dPCR, using primers Ams06KbF and Ams189R. For v-dPCR, sample sub-aliquots were treated with PMA, PMAxx, and combinations of both dyes with SDS (+SDS) or PMA Enhancer (+E). Culturable cell counts were performed by spread plate on SNAN and CCT media. Three cankers were analyzed per time period. Each column represents mean values of total, viable or culturable cell counts of three different cankers, with error bars indicating the SD.

In apple cankers, *E. amylovora* total cell numbers in mid-summer (Fig. 9a) were about one log unit higher than in mid-winter (p < 0.0001) (Fig. 9b). Live cell counts were about 2 log units lower than the total ones, regardless of the time period or the viability-staining treatment assayed, with differences between total and viable or culturable cell quantification values being very significant (*p* < 0.0001) (Fig. 9). No differences were detected between the v-dPCR viable cell counts performed using PMA and PMAxx, regardless of the time period assayed or their combination with SDS or PMA Enhancer (*p* > 0.05). In most cases, the addition of SDS or PMA Enhancer reduced viable cell counts compared to the control treatments with PMA or PMAxx alone (Fig. 9a,b), although these differences were statistically significant only in the case of mid-summer apple cankers treated with PMA + E (*p* < 0.05) (Fig. 9a).

*E. amylovora* culturable counts on CCT and SNAN were similar in mid-summer cankers (*p* > 0.05), but in the mid-winter cankers, a lower *E. amylovora* recovery on CCT compared to SNAN was observed (*p* < 0.01) (Fig. 9b). CCT contains crystal violet and other selective compounds^47^ which show low toxicity for non-stressed *E. amylovora* cells. Poor recovery efficiencies on selective media as compared to general media are usually linked to injured bacteria which become more sensitive to the selective compounds of the medium^48^. Accordingly, another advantage of v-dPCR is the detection of live cells regardless of their ability to form colonies on solid media. This might be especially important for detecting cells in the VBNC state. This survival strategy has been reported in more than 100 bacterial species^12^ including *E. amylovora* and other important plant pathogens, which represents a challenge for their management.

**Figure 10:**
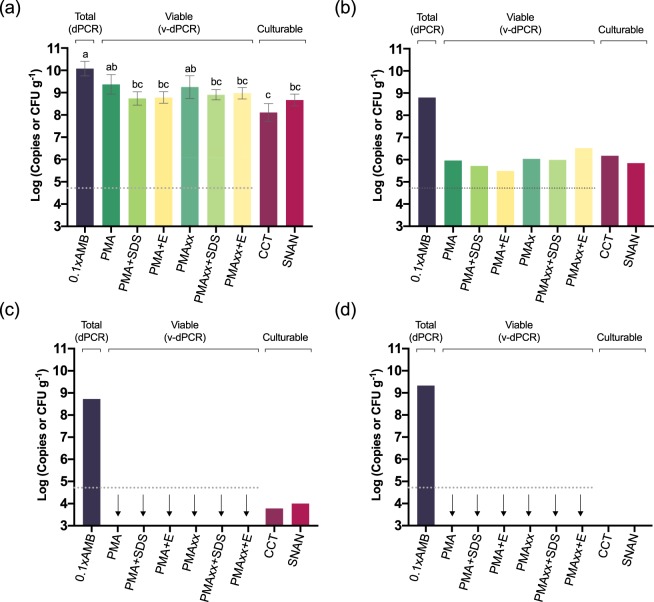
*E. amylovora* total, live and culturable cell populations in fire blight cankers on pear. Fire blight infections originated in early-spring of 2016, and cankers collected in mid-summer (July 2016) (**a**) and mid-winter (January 2017) (**b**–**d**). For sample analysis, cankers were homogenized with 0.1xAMB. Total cell counts were performed by DNA extraction and dPCR. For v-dPCR, samples were treated with PMA, PMAxx, or any of these dyes combined with SDS (+SDS) or PMA Enhancer (+E). In both cases, dPCR was performed using primers Ams06KbF/Ams189R and the probe Ams141T. Culturable cell counts were performed by spread plate on SNAN and CCT media. In cankers collected in mid-summer, *E. amylovora* quantification values were similar, so each column represents mean values of total, viable and culturable cell counts of three different cankers, with error bars indicating the SD (**a**). In cankers collected in mid-winter, substantial differences among cankers in *E. amylovora* populations were observed. Separate graphs were used to highlight differences among cankers (**b**,**c**,**d**). Dashed lines represent the limit of detection and quantification of dPCR. Arrows represent viable cell counts equal to 0 or below the dPCR detection limit. The absence of columns corresponding to plate counts represent values below the detection limit of the technique, which is shown in graphs as the minimum value at the Y axis.

The *E. amylovora* cell counts in pear cankers harvested in mid-summer (Fig. 10a) were also higher than in those sampled in mid-winter (p < 0.0001) (Fig. 10b–d). The differences between total and viable cell numbers in pear cankers collected in mid-summer (about 0.5 log units) were smaller than those in apple at the same time period (ca. 2 log units) (Fig. 9). This reflects a greater number of *E. amylovora* dead cells in apple than in pear cankers, which might be linked to the higher resistance of apple to fire blight in comparison to pear. V-dPCR data revealed no differences between PMA- and PMAxx-treated samples (*p* > 0.05), or between the samples treated with SDS or PMA Enhancer compared to their counterparts treated only with PMA or PMAxx (*p* > 0.05) (Fig. 10a). Similarly, no statistically significant differences were detected between plate counts carried out on CCT and SNAN (p > 0.05) (Fig. 10a).

In pear cankers harvested in mid-winter, total cell numbers also showed similar values in all of the analyzed samples, although culturable and viable cell counts significantly differed among cankers (Fig. 10b–d). In only one of the three cankers collected in mid-winter (Fig. 10b) were *E. amylovora* viable cell populations at the levels similar to those in apple cankers collected in the same time period (Fig. 9b). In the remaining cankers (Fig. 10c,d), the number of viable cells fell below the detection limit of the technique under our experimental conditions (ca. 10^3^ CFU mL^−1^ of plant macerates, or about 5 × 10^4^ CFU g^−1^ of canker). This coincided with *E. amylovora* culturable cell counts below the v-dPCR detection limit (ca. 10^4^ CFU g^−1^) (Fig. 10c,d) or no detection of culturable bacteria on any of the assayed media (Fig. 10d) (limit of detection of plate counts: 20 CFU mL^−1^ of plant macerates or 10^3^ CFU g^−1^ of canker).

In both apple and pear cankers, v-dPCR provided viable cell counts similar to those on SNAN with most of the assayed PMA treatments (Fig. 9a,b), indicating that most of the viable cells in cankers are in a culturable state. In mid-summer apple samples that were treated with PMA + E, a small decrease of viable versus culturable cell counts was observed (*p* < 0.05) (Fig. 9a), although these differences were not detected in apple cankers collected in winter or in pear cankers in any of the assayed periods (Figs 9 and 10).

Unlike in previous assays where the use of PMA Enhancer or SDS was required to eliminate false-positive signals (Fig. 6), the analysis of natural cankers revealed a good suppression of dead-cell DNA amplification, even in samples treated with PMA or PMAxx alone (i.e. v-dPCR live cell counts were similar to those of culturable cells in most of the analyzed samples) (Figs 9 and 10). The differences between *in vitro* and natural sample analysis are probably related to conditions in cankers favoring a more efficient degradation of dead cell membranes compared to the killing method employed in this work. Moreover, the analysis of pear cankers harvested in mid-winter revealed the complete elimination of false-positive signals in samples containing at least 100,000 times more dead cells than live ones (i.e. ca. 10^9^ total cells g^−1^ from which only about 10^4^ cells or less were culturable) (Fig. 10c,d). These results show the efficiency of most of the tested treatments to analyze *E. amylovora* viable cell populations in cankers. Accordingly, based on our data, the suggested protocol to perform *E. amylovora* viable-cell analysis of cankers by v-dPCR consists of the following steps: *i)* Sample processing with 0.1xAMB, in a ratio 1:50 (w/v); *ii)* treatment of samples with PMAxx, PMAxx + E or PMAxx + SDS; *iii)* dPCR using primers Ams06KbF/Ams189R. Detailed information on each step can be found in the Methods section and a detailed protocol for the analysis of *E. amylovora* total, viable, and culturable cell populations in cankers is provided as supplementary information (Fig. S5).

Overall, our results reveal the valuable potential of v-dPCR and the combined analysis of total, viable, and culturable *E. amylovora* populations in cankers to unveil cryptic stages of the *E. amylovora* life cycle or specific aspects linked to host-pathogen interactions. Although preliminary, canker analysis revealed that winter weather conditions and/or the cessation of the host’s vegetative growth are probable factors inducing the general decline of *E. amylovora* live cell populations in winter, particularly on pear. Our results also correlate well with the hypothesis that the pathogen overwinters in only a certain percentage of cankers^1,3^. Differences between bacterial cell numbers in apple versus pear cankers might indicate a host- and/or bacterial strain-dependent survival of *E. amylovora*. More research on these topics will provide more information on *E. amylovora* cell population dynamics in cankers and its relationship with environmental and/or host-related factors, as well as on establishing links between host resistance and *E. amylovora* survival in cankers. Our protocol might also be useful for data generation to improve the accuracy of fire blight forecasting systems, which currently assume abundance of primary bacterial inoculum every year for disease renewal^49^.

## Methods

### Bacterial strains and culture media

Unless stated otherwise, the *E. amylovora* strain ATCC 49946 was used in all the experiments. Other *E. amylovora* strains and/or plant pathogenic bacterial species employed in this work are summarized in the Supplementary Table S3.

Bacterial cultures were cryopreserved at −80 °C in 20% glycerol (v/v). All the strains were grown at 28 °C in/on the general media Luria-Bertani^50^ (LB) and/or Sucrose Nutrient Agar^51^ (SNA). *E. amylovora* isolation and plate counts from natural samples were carried out on SNA amended with 21.6 mg L^−1^ natamycin (Amtech Biotech Co. Ltd., Qiqihar, China) (SNAN) and/or on Crystal violet-Cycloheximide-Thallium nitrate (CCT) medium^48^.

### *E. amylovora-*free plant material, sources and obtaining of plant macerates

All the *E. amylovora-*free plant material was collected from orchards at the Cornell University’s Hudson Valley Research Laboratory (HVRL) in Highland (NY, USA). 1-year-old apple ‘Honeycrisp’ and pear ‘Bosc’ branches were harvested, cut in 15-cm-long pieces and stored at −80 °C after fast freezing in liquid N_2_ until use.

To obtain *E. amylovora* free plant macerates, branch pieces were left to thaw at room temperature for 10 min. Slices from the branch surface containing bark and vascular cambium were excised with a sterile scalpel, weighed, placed into a Ziploc plastic bag containing either ice-cold AMB^38,39^ or 0.1xAMB in 10 mM phosphate buffered saline (PBS) pH 7.4, and homogenized by crushing with a hammer. The volume of maceration buffer in each bag was proportional to the sample weight, in a ratio 1:50 (w/v). Plant macerates were freshly prepared before each experiment, and chilled on ice until use. The absence of *E. amylovora* in this plant material was confirmed by microbiological and molecular methods, according the International Standard for Phytosanitary Measures 27 (ISPM 27, Annex 13) for *E. amylovora* detection in asymptomatic plant material^39^.

### Preparation of *E. amylovora* live and/or dead cell stocks

In most experiments, *E. amylovora-*free plant macerates were inoculated with either *E. amylovora* live, dead or a mixture of live and dead cells. Live cell suspensions were prepared with overnight cultures (16 h) in LB, by centrifugation and resuspension of the pellets in sterile distilled water (diH_2_O) thrice.

Unless otherwise specified, dead cells were obtained by heat-treatment, exposing 1 mL of *E. amylovora* live cell suspensions in diH_2_O to 85 °C for 30 min. In both cases, the OD_600_ nm of the cell stocks was adjusted to 1 or 0.1 (10^9^ or 10^8^ CFU mL^−1^, respectively) and employed to inoculate plant macerates at the desired concentrations, either with the stocks or with their serial dilutions in diH_2_O.

Live cell concentrations in stocks were confirmed by drop-plate on LB agar, as described elsewhere^52^. The absence of live cells in heat-treated bacterial suspensions was confirmed by negative growth on LB agar after spread-plating 0.1 mL of the challenged cell suspension in triplicate and incubation for 72 h at 28 °C.

All the experiments including *E. amylovora-*free plant macerates included negative controls, to ensure the absence of *E. amylovora* cells prior to sample inoculation.

### DNA extractions

Before dPCR, 0.5 mL aliquots from plant macerates, treated or not with the viability-PCR dye PMA (see below), were subjected to DNA extraction using DNeasy Plant Mini Kit (Qiagen, Hilden, Germany) according to the manufacturer instructions and resuspending DNA in a final volume of 200 μL. When required, the same kit was also used for bacterial genomic DNA extractions, starting from 0.5 mL overnight cultures in LB. Samples were stored at −20 °C until use.

### dPCR conditions

dPCR was performed with the QS3D dPCR System (ThermoFisher Scientific). Each dPCR reaction was prepared in a final volume of 16 μL, composed of 8 μL of QS3D dPCR Master Mix v2 (Applied Biosystems, Frederick, MD, USA), 0.8 μL of 20x primers/probe mix (final concentrations, 0.9 and 0.2 μM, respectively) (Tables 1), 6.4 μL of direct or diluted DNA sample and 0.8 μL of nuclease free water. The QS3D dPCR chips were loaded with 15 μL reaction mix by using the QS3D dPCR automatic loader, and the dPCR amplification was conducted with a GeneAmp 9700 PCR thermocycler (Applied Biosciences, Foster City, CA, USA). Amplification conditions were: 10 min at 95 °C for DNA polymerase activation, followed by 39 two-step cycles of 1 min at 60 °C and 15 sec. 98 °C, and a final extension at 60 °C for 2 min. The annealing temperature, and primer/probe concentrations were chosen based on previous works^19,31^. One-off assays using higher and lower annealing temperatures led to a smaller separation between background and positive fluorescence signals, so we continued using 60 °C with all primer combinations.

After DNA amplification, the chips were transferred to the QS3D Instrument for imaging, and end-point fluorescence data were collected and analyzed with the QS3D AnalysisSuite Cloud Software (version 3.1.2-PRC-build-03, Thermo Fisher Scientific) under the Absolute Quantification module, maintaining automatic settings. The global threshold was determined automatically by the software, and adjusted manually when required using the signals observed in no-template controls as a reference. Output data provided by the software included the mean number of copies per reaction, the number of copies per μL, the number of partitions qualified for quantification, the number of partitions negative for target DNA amplification, along with other parameters related to quantification and the quality assessment of the analysis. Only chips with more than 15,000 out of 20,000 partitions qualifying for quantification were used for analysis.

Discrimination between positive and negative fluorescence calls was based on the QS3D Analysis Suite output data (Thermo Fisher Scientific). Signals of no-template controls and those from DNA extractions corresponding to *E. amylovora-*free plant material, from both apple and pear, as well as positive controls with different *E. amylovora* DNA concentrations were used to discriminate background fluorescence from positive calls. Based on these experiments, only chips with a number of positive calls higher than 5 were considered positive for *E. amylovora* detection.

Quantitative data provided by the QS3D Analysis Suite Cloud Software was used to calculate the number of target copies per g of canker tissue, with the formula:

$$Copies\,{g}^{-1}=Copies\,\mu {L}_{{\rm{Rxn}}}^{-1}\times \frac{1}{{D}_{{\rm{Rxn}}{\rm{tube}}}}\times \frac{1}{{D}_{{\rm{DNA}}}}\times \frac{1}{{E}_{{\rm{DNA}}{\rm{extr}}}}\times \frac{Vo{l}_{{\rm{Elution}}(\mu L)}}{Vo{l}_{{\rm{Pl}}{\rm{.}}{\rm{Macerates}}({\rm{mL}})}}\times \frac{1}{Rati{o}_{\mathrm{Canker}(g):\mathrm{AMB}(\mathrm{mL})}}$$

where *Copies μL*^−1^_Rxn_ are the copies of target DNA per μL in the reaction tube, provided by the QS3D Analysis Suite software; *D*_Rxn tube_ is the dilution of sample DNA in the dPCR reaction tube (e.g. 6.4 μL sample DNA in 16 μL of dPCR mastermix); *D*_DNA_ is an optional dilution of sample DNA before dPCR (e.g. typically, 1/50–1/100 dilutions of sample DNA were required for the analysis of total cell quantification in natural canker samples); *E*_DNA extr_ is the efficiency of the DNA extraction with the kit (in our case, a 27.9% for apple, and a 10.4% for pear tissues); *Vol*_Elution (μL)_ is the final elution volume of sample DNA after extraction with the kit (in our case, 200 μL); *Vol*_Pl_. _Macerates (mL)_ is the volume of plant macerates used for DNA extraction (usually, 0.5 mL); the *Ratio*_Canker:AMB_ was taken into consideration to calculate the concentration of *E. amylovora* cells per gram of analyzed canker (ratio canker:AMB, 1:50).

Other parameters related to the dPCR performance such as the quantity of target DNA copies per reaction well, the total number of reaction wells in the chip that exceed the selected quality threshold, or the number of positive calls for FAM dye in the chip were also provided by the software, and are specified in tables within the text or as supplementary information (Supplementary Tables S1, S2, S5), according to the digital MIQE guidelines^53^.

### Digital PCR using a transferred protocol for qPCR

A preliminary evaluation of the QS3D dPCR performance and robustness (capacity of the method to remain unaffected by variations in sample composition) was performed before testing the technique for the analysis of *E. amylovora* live cell populations in cankers.

*E. amylovora-*free apple and pear macerates obtained using AMB were inoculated with strains ATCC 49946 and CFBP 1430 at final concentrations ranging from 10^2^ to 10^8^ CFU mL^−1^. After DNA extraction, samples were analyzed by dPCR using the same primers, probe and annealing temperature of an already existing qPCR protocol^31^ which had previously successfully been used with a different dPCR platform^19^. Data from three independent experiments were log-transformed and the correlation between dPCR counts of target DNA copies and the inoculated CFUs mL^−1^ was analyzed by linear regression analysis, using GraphPad Prism 8 (version 8.1.2). The same software was used for the statistical analysis of the remaining experiments in this work.

### PMA treatment

The PMA treatment was designed based on Fittipaldi *et al*.^30^ and works analyzing plant material^27^ or more complex samples^26^. Unless otherwise stated, 0.4–0.5 mL plant macerates were treated with PMA (Biotium Inc., CA, USA) at a final concentration of 100 μM, in 2 mL tubes and incubated in the dark for 5 min with shaking (150 rpm). Tubes were then placed horizontally on ice, and exposed to light from two halogen bulbs (500 W each) at a distance of 20 cm from the light source, as described elsewhere^26,27^. In other works, the usual times for PMA photolysis range from 2 to 20 min^30^. In our case, light exposure periods above 5 min were enough to provide consistent results among sample replicates. Hence, a standard 10-min PMA photo-activation was applied in all the experiments. For natural canker analysis, a commercial, improved version of PMA, known as PMAxx (Biotium Inc., CA, USA), was also used the same as described for PMA. After the PMA (or PMAxx) treatment, samples were centrifuged at 13,000 rpm for 10 min, the supernatant discarded and pellets stored at −80 °C until use.

### Correlation between v-dPCR and culturable cell counts

Apple macerates were prepared in AMB and inoculated with live cells ranging from 10^3^ to 10^6^ CFU mL^−1^. Samples were sub-aliquoted and subjected to v-dPCR after PMA treatment. Aliquots of the same samples non-treated with PMA were used as controls, to quantify total target DNA copies by dPCR. The experiment was repeated in three independent assays. Correlation between v-dPCR and the inoculated CFUs was determined by linear regression analysis.

### Correlation between v-dPCR and culturable cell counts in the presence of dead cells

*E. amylovora-*free plant macerates were prepared with AMB (in initial analyses) or 0.1xAMB, and inoculated with 10^6^ *E. amylovora* dead cells mL^−1^. Aliquots of these dead cell suspensions were additionally inoculated with increasing live cell concentrations ranging from 10^3^ to 10^6^ CFU mL^−1^. Samples were sub-aliquoted in two parts. One part was treated with PMA, to calculate viable cell concentrations, and the other part was directly subjected to dPCR, to determine total cell counts. The experiment was repeated in three independent assays, and coefficients of correlation between v-dPCR and culturable cell counts were determined by linear regression analysis.

### Effect of PMA concentration and the *E. amylovora* killing method on the exclusion of dead-cell signals by v-dPCR

PMA concentrations ranging 100–500 μM were used to stain aliquots of apple plant macerates prepared in AMB and inoculated with 10^6^ *E. amylovora* dead cells mL^−1^. Samples non-treated with PMA were used as controls. The assay was performed in triplicate. Mean differences between v-dPCR values for each PMA concentration were calculated after log-transformation of data, by ordinary one-way ANOVA and a Tukey’s multiple comparison test.

To detect a possible effect of the killing method on the suppression of false-positive signals by v-dPCR, apple branch macerates prepared in AMB were inoculated with *E. amylovora* live cells (10^3^ CFU mL^−1^) mixed with dead cells (10^6^ cell mL^−1^) obtained either by heating at 85 °C for 30 min (standard killing method used in this work), heating at 57 °C for 18 h, or exposure to 70% isopropanol for 10 min. Samples were then sub-aliquoted and analyzed in parallel by dPCR, v-dPCR (regular treatment with 100 μM PMA) and plate counts. The experiment was performed in triplicate. The differences between log-transformed viable (v-dPCR) and culturable cell counts for each killing method were calculated, and compared to the control by a Kruskal-Wallis with Dunn’s *post hoc* test.

### Effect of the thermal cycle number and the amplicon length on false-positive signal suppression and live cell population discrimination by v-dPCR

Apple branch macerates prepared in AMB were inoculated either with *E. amylovora* at 7 × 10^6^ CFU mL^−1^ (live cell control), or live/dead cell mixtures containing a 10%, a 1%, or a 0% of live cells with respect to the live cell control, and a constant background of 7 × 10^6^ dead cells mL^−1^. For v-dPCR, samples were treated with PMA and dPCR was performed under different conditions: (i) using the same primers and probe as Pirc *et al*.^31^, with 40 amplification cycles; (ii) Pirc *et al*.^31^ primers, but reducing the number of thermal cycles to 30; (iii) or a newly designed primer combination targeting a 966 bp DNA sequence (Ams09KbF/Ams189R) (Table 1), and 40 amplification cycles. The assay was performed in two biological repeats. For an easier comparison of theoretical (100%, 10%, 1%, 0%) and obtained live cell percentage values, viable cell concentrations in the control and in live/dead cell mixtures were expressed as percentages with respect to the live cell control. Differences between fluorescence peaks of negative (background signal) and positive calls (positive detection of target DNA) by dPCR were monitored through the QS3D Analysis Suite.

### Primer design and effect of increasing amplicon lengths on the exclusion of dead-cell signals by v-dPCR

To obtain different amplicon lengths, the reverse primer Ams189R, from a previous publication^31^ was combined either with the original primer Ams116F^31^ or with newly designed primers binding DNA upstream of the reverse primer sequence (Table 1). Primers were designed with Primer-BLAST (https://www.ncbi.nlm.nih.gov/tools/primer-blast/), using the *amsC* gene sequence of *E. amylovora* ATCC 49946 plus additional 500 bp upstream of the reverse primer sequence as template. All primers were designed using the web application default settings, including an optimal melting temperature of 60 ± 3 °C, a G + C content between 20–80% and primer sizes of 20 ± 5 nucleotides.

Prior to primer utilization for dPCR, the size of the amplicons with each primer pair was confirmed by classical PCR. Although signal generation by dPCR is less dependent on highly efficient assays than qPCR^53^, a gradient PCR was performed with all primer pairs to identify possible differences in the efficiency of PCR amplification at different temperatures. All primers showed similar optimal annealing temperatures. Also, preliminary assays on dPCR showed 60 °C as the optimal temperature for both the original primers from the work of Pirc *et al*.^31^, and the primers designed in this work. Temperatures above and below 60 °C led to a worse separation between background fluorescence signals and the ones corresponding to positive detection of target DNA. None of the assayed primers showed signs of unspecific amplification either by classical PCR (no unspecific bands) or by dPCR (no more than 2 clusters of fluorescence signals, corresponding to negative and positive amplification) after 40 DNA amplification cycles.

Primer specificity for the *E. amylovora* target DNA was tested using genomic DNA extractions of a collection of *E. amylovora* strains, including reference strains, natural isolates, and other plant pathogenic and saprophytic bacterial species listed in the Supplementary Table S3.

To determine the effect of the amplicon length on v-dPCR live/dead cell discrimination, apple branch macerates prepared in AMB were artificially inoculated with either *E. amylovora* live (10^6^ CFU mL^−1^), dead (10^6^ dead cells mL^−1^) or a defined ratio of live:dead cells (10^3^:10^6^ cells mL^−1^). Each sample was sub-aliquoted for total and live cell quantification. The dPCR reactions were performed with primer combinations targeting a 74, 325, 627 and 966 bp DNA sequence, respectively (Table 1). The assay was repeated in three independent experiments. The effect of the amplicon size on total (dPCR) or live (v-dPCR) cell quantification in samples composed of live, dead or a live/dead cell mixture was analyzed by two-way ANOVA with Tukey’s multiple comparisons test.

### Effect of the maceration buffer and the addition of SDS or PMA Enhancer on dead-cell signal suppression by v-dPCR

Apple branch macerates prepared either in AMB or in 0.1xAMB were inoculated with either *E. amylovora* live (10^6^ CFU mL^−1^), dead (10^6^ dead cell mL^−1^), or mixed live (10^3^ CFU mL^−1^) and dead (10^6^ dead cell mL^−1^) cells as mentioned above. Each sample was sub-aliquoted and subjected to: (i) dPCR to quantify total cells; (ii) treatment with PMA; (iii) PMA plus 0.0025% SDS (w/v) (PMA + SDS); (iv) PMA plus 1x PMA Enhancer for Gram-negative bacteria (Biotium Inc., CA, USA) (PMA + E). Based on the results from the previous experiment, samples were subjected to DNA extraction and dPCR, using the primers targeting the 627 bp DNA sequence.

The SDS concentration was selected based on a minimal inhibitory concentration (MIC) assay performed in fresh LB broth inoculated with a 1/100 dilution of an overnight culture of *E. amylovora* ATCC 49946 in the same medium, and amended with increasing two-fold concentrations of SDS ranging from 0 to 0.32% (w/v). The greatest SDS concentration not having a measurable effect on the OD_600_ nm after a 16-h incubation period was chosen to determine the effect of SDS on the selective discrimination of live cells during PMA treatments.

These assays were performed in triplicate, in two independent experiments. After log-transformation of data, a two-way ANOVA with Sidak’s multiple comparisons tests was conducted to determine the main effects of the maceration buffer and the PMA treatment.

### dPCR performance using primers Ams06KbF and Ams189R

Similar to the preliminary assay using the primers of Pirc *et al*.^31^, the dPCR performance with the primers Ams06KbF/Ams189R was characterized using apple and pear plant macerates prepared in 0.1xAMB and inoculated with increasing *E. amylovora* concentrations ranging from 10^3^ to 10^7^ CFU mL^−1^. Data from two independent experiments performed in triplicate were used to determine the correlation between dPCR values and the inoculated CFUs mL^−1^ by linear regression analysis. The assay variability (%CV) for each of the tested bacterial concentrations was calculated by dividing the SD by the mean of the replicates, multiplied by 100. The dynamic range of the technique, as well as the linear relationship and correlation coefficient (R^2^) for dPCR values and culturable cell counts were determined after log-transformation of data. The limits of detection (the lowest concentration at which all replicates are positive for detection) and quantification (the lowest concentration showing positive detection in all the replicates with a %CV equal or below 25%) were defined based on Pavšič *et al*.^54^.

### Selective live cell discrimination in mixed live/dead cell populations by optimized plant material processing and v-dPCR conditions

The v-dPCR selective detection of *E. amylovora* viable cells in the presence of dead cells, as well as the correlation of v-dPCR values to the inoculated CFUs mL^−1^ was tested in apple and pear plant macerates homogenized in 0.1xAMB and inoculated with increasing live cell concentrations (from 10^3^ to 10^6^ CFU mL^−1^) with a constant background of 10^6^ dead cell mL^−1^. Total cell counts were performed by dPCR. For viable cell counts, samples were treated with PMA plus 1x PMA Enhancer before dPCR. In all cases, dPCR was performed using primers Ams06KbF and Ams189R. Assays were performed in triplicate and repeated twice, and the linear regression best fit through all the v-dPCR dataset points was calculated with GraphPad Prism 8.1.2 (227).

### Canker sampling and analysis of *E. amylovora* total, live and culturable cell populations

A total of 12 natural fire blight apple (cv. Honeycrisp) and pear (cv. Bosc) cankers were collected from orchards in Peru (NY, USA) and at the HVRL experimental station in Highland (NY, USA), respectively. In both cases, fire blight infections started in early spring of 2016, canker formation was monitored through time, and samples were collected in mid-summer (July 2016) and mid-winter (January 2017). All the sampled cankers had defined margins. Branch diameters and other data related to canker characteristics are detailed in the Supplementary Table S4.

For canker analysis, tissue slices containing the bark and the vascular cambium below the bark were excised from the canker area plus a 4 mm wide ring surrounding the canker margin, using a sterile scalpel. The sliced samples were weighed, transferred to individual plastic bags, mixed in a ratio 1:50 (w/v) with 0.1xAMB, and homogenized with a hammer, as described above.

Afterward, canker macerates were aliquoted to perform (i) plate counts on SNAN and CCT by spread plating 50 μL after serial tenfold dilutions of samples in 10 mM PBS pH 7.4; (ii) total cell quantification by dPCR; (iii) and v-dPCR after treatment with PMA, PMAxx, or combinations of any of these two viability PCR dyes with 0.0025% SDS (final concentration) or 1x PMA Enhancer for Gram-negative bacteria, according to the manufacturer’s instructions.

All dPCR reactions were run using the primer pair and probe Ams06KbF/Ams189R and Ams141T, respectively (Table 1). When required, samples for dPCR and v-dPCR were diluted from tenfold up to 500-fold to reach target copy concentrations inside of the dPCR quantification range.

In apple cankers, a strong consistency between the total, viable and culturable cell counts was observed among the samples collected in the same time period. A similar phenomenon was observed in pear cankers collected in mid-summer. However, the mid-winter cankers revealed very different *E. amylovora* population patterns depending on the analyzed sample. For a more accurate statistical analysis to assess differences among viability-staining treatments, consistent data among samples harvested in the same time period were grouped together (Figs 9a,b and 10a), while mid-winter cankers differing in the *E. amylovora* population content were represented separately to illustrate specific aspects of the v-dPCR efficiency as well as other important insights tied to pathogen biology.

## Supplementary information

Supplementary Information

## Data Availability

Data supporting the findings of this study are available within the publication, as supplementary material and/or from the corresponding authors on request.
