# Publisher Correction: Development of a viability digital PCR protocol for the selective detection and quantification of live *Erwinia amylovora* cells in cankers

**DOI:** 10.1038/s41598-019-49873-9

**Published:** 2019-10-29

**Authors:** Ricardo D. Santander, Christopher L. Meredith, Srđan G. Aćimović

**Affiliations:** Plant Pathology and Plant-Microbe Biology Section, Cornell University, Hudson Valley Research Laboratory, Highland, NY USA

Correction to: *Scientific Reports* 10.1038/s41598-019-47976-x, published online 08 August 2019

In the original version of the Article, the full error bars were omitted from Figure 9A due a technical error during publication.

In addition, in the Results and Discussion Section under the subheading ‘Direct transfer of a qPCR protocol to the QS3D dPCR system’,

“A known qPCR protocol for *E*. amylovora^31^ was transferred to the QS3D dPCR platform, using the same primer and probe concentrations and thermal cycling conditions as a first step to test the QS3D dPCR technology.”

now reads:

“A known qPCR protocol for *E*. *amylovora*^31^ was transferred to the QS3D dPCR platform, using the same primer and probe concentrations and thermal cycling conditions as a first step to test the QS3D dPCR technology.”

Finally, in the Legend of Table 2,

“^c^Log Copies mL^−1^ = Log [copies μL^−1^_rxn_ × 1/D_rxn tube_ x1/E_DNA Extr_. X1/Vol_Pl.Macerates_]; i.e. Log (copies μL^−1^_rxn_ × 1.79 × 10^3^) for Apple, and Log (copies μL^−1^_rxn_ × 4.81 × 10^3^) for pear.”

now reads:

“^c^Log Copies mL^−1^ = Log [copies μL^−1^_rxn_ × 1/D_rxn tube_ × 1/E_DNA Extr_ × 1/Vol_Pl.Macerates_]; i.e. Log (copies μL^−1^_rxn_ × 1.79 × 10^3^) for Apple, and Log (copies μL^−1^_rxn_ × 4.81 × 10^3^) for pear.”

This has been corrected in both the PDF and HTML versions of the Article.

