# Supplementary material for: Development of a viability digital PCR protocol for the selective detection and quantification of live Erwinia amylovora cells in cankers
Source: Sci Rep. 2019 Aug 8;9:11530. doi: 10.1038/s41598-019-47976-x (PMC6687816; doi:10.1038/s41598-019-47976-x)
Supplement: Supplementary file 1 — Supplementary Information [file 41598_2019_47976_MOESM1_ESM.docx]

**Supplementary Information**

**Title**

Development of a viability digital PCR protocol for the selective detection and quantification of live *Erwinia amylovora* cells in cankers

**Authors**

Ricardo Delgado Santander, Christopher Meredith, Srđan G. Aćimović*

**Institution**

Plant Pathology and Plant-Microbe Biology Section, Cornell University, Hudson Valley Research Laboratory, Highland, NY, USA.

***Corresponding Author**

Srđan G Aćimović

e-mail: [acimovic@cornell.edu](mailto:acimovic@cornell.edu)

Phone: +1 845 691 7231

**Supplementary Tables**

**Table. S1.**Transference of a qPCR protocol to the QS3D dPCR system for the absolute quantification of *E. amylovora* ATCC 49946 cell numbers in plant material.

| **Host** | **Log CFU mL^-1 b^** | **Log Copies mL^-1^** **(± SD)^c^** | **Copies rxn^-1^ (± SD)^c,d^** | **# Qualified by QT (± SD)^c,e^** | **# Pos (± SD)^c,f^** |
| --- | --- | --- | --- | --- | --- |
| **Apple cv Honeycrisp ^a^** | 8.50 | 7.37 ± 0.13 | 7.451 ± 1.819 | 15374 ± 506 | 15797 ± 861 |
|  | 7.50 | 7.28 ± 0.10 | 8.053 ± 1.242 | 16098 ± 1568 | 16082 ± 1573 |
|  | 6.50 | 6.68 ± 0.07 | 2.003 ± 0.014 | 15935 ± 1060 | 13743 ± 912 |
|  | 5.50 | 5.77 ± 0.05 | 0.248 ± 0.032 | 17058 ± 1059 | 3071 ± 1375 |
|  | 4.50 | 4.73 ± 0.00 | 0.023 ± 0.004 | 17190 ± 507 | 384 ± 70 |
|  | 3.50 | 3.81 ± 0.07 | 0.003 ± 0.001 | 17289 ± 672 | 53 ± 21 |
|  | 2.50 | 2.09 ± NA | 0.001 ± NA | 16628 ± 1443 | 5 ± 3 |
|  |  |  |  |  |  |
| **Pear cv. Bosc ^a^** | 8.50 | 7.69 ± 0.09 | 7.757 ± 1.426 | 17787 ± 469 | 17767 ± 482 |
|  | 7.50 | 7.62 ± 0.04 | 6.589 ± 0.577 | 17507 ± 392 | 17457 ± 412 |
|  | 6.50 | 6.69 ± 0.03 | 0.780 ± 0.059 | 17319 ± 1032 | 9364 ± 1019 |
|  | 5.50 | 5.65 ± 0.02 | 0.071 ± 0.003 | 17625 ± 478 | 983 ± 554 |
|  | 4.50 | 4.54 ± 0.06 | 0.005 ± 0.001 | 17874 ± 202 | 50 ± 4 |
|  | 3.50 | 3.67 ± 0.87 | 0.001 ± 0.000 | 17032 ± 1326 | 11 ± 4 |
|  | 2.50 | 0.00 ± NA | 0.000 ± NA | 18294 ± 583 | 3 ± 2 |

^a^Apple and pear branch macerates were obtained by crushing plant material in a plastic bag containing AMB in a ratio 1:50 (w/v). Data are mean values from three biological repeats. SD, standard deviation.

^b^ Data shown in this column correspond to average bacterial concentrations of three different *E. amylovora* stocks, employed to inoculate samples in three independent assays. Values in each dilution were calculated based on the stock concentrations. Hence, the SD in all cases was the same, ± 0.13.

^c^ Average values of three biological repeats. In some cases, the SD could not be calculated because only 1 or none of the analyzed samples were positive for *E. amylovora* detection (NA, not applicable).

^d^ Quantity of target DNA copies per reaction well.

^e^ Total number of reaction wells in the chip that exceed the selected quality threshold.

^f^ Total number of positive calls for FAM dye in the chip, as determined from the Review Calls scatter plot. Based on the no-template control and plant material negative control analysis, chips with 5 or less than 5 positive calls were considered negative for *E. amylovora* detection, and the provided quantification values were not considered.

**Table. S2.**Transference of a qPCR protocol to the QS3D dPCR system for the absolute quantification of *E. amylovora* CFBP 1430 cell numbers in plant material.

| **Host** | **Log CFU mL^-1 b^** | **Copies mL^-1^ (± SD)^c^** | **Copies rxn^-1^ (± SD) ^c,d^** | **# Qualified by QT (± SD) ^c,e^** | **# Pos. (± SD) ^c,f^** |
| --- | --- | --- | --- | --- | --- |
| **Apple cv Honeycrisp ^a^** | 8.44 | 7.23 ± 0.09 | 7.176 ± 0.965 | 17261 ± 876 | 17237 ± 855 |
|  | 7.44 | 7.31 ± 0.02 | 8.502 ± 0.366 | 17395 ± 89 | 17391 ± 91 |
|  | 6.44 | 6.42 ± 0.98 | 1.115 ± 0.210 | 16600 ± 949 | 11530 ± 2041 |
|  | 5.44 | 5.55 ± 0.05 | 0.149 ± 0.017 | 16711 ± 1221 | 2298 ± 76 |
|  | 4.44 | 4.51 ± 0.08 | 0.014 ± 0.003 | 17439 ± 276 | 237 ± 42 |
|  | 3.44 | 3.62 ± 0.10 | 0.002 ± 0.000 | 15670 ± 1091 | 27 ± 9 |
|  | 2.44 | 2.06 ± 0.23 | 0.000 ± 0.000 | 16956 ± 403 | 6 ± 4 |
|  |  |  |  |  |  |
| **Pear cv. Bosc ^a^** | 8.44 | 7.65 ± 0.17 | 7.079 ± 1.051 | 15921 ± 579 | 15893 ± 568 |
|  | 7.44 | 7.13 ± 0.08 | 2.164 ± 0.379 | 16329 ± 690 | 14313 ± 991 |
|  | 6.44 | 6.43 ± 0.07 | 0.425 ± 0.062 | 17973 ± 249 | 6204 ± 735 |
|  | 5.44 | 5.47 ± 0.05 | 0.045 ± 0.005 | 17168 ± 937 | 821 ± 220 |
|  | 4.44 | 4.55 ± 0.09 | 0.006 ± 0.001 | 16748 ± 582 | 94 ± 18 |
|  | 3.44 | 3.63 ± 0.12 | 0.001 ± 0.000 | 17815 ± 281 | 12 ± 3 |
|  | 2.44 | 2.49 ± NA | 0.000 ± NA | 18020 ± 508 | 5 ± 3 |

^a^Apple and pear branch macerates were obtained by crushing plant material in a plastic bag containing AMB in a ratio 1:50 (w/v). Data are mean values from three biological repeats. SD, standard deviation.

^b^ Data shown in this column correspond to average bacterial concentrations of three different *E. amylovora* stocks, employed to inoculate samples in three independent assays. Values in each dilution were calculated based on the stock concentrations. Hence, the SD in all cases was the same, ± 0.13.

^d^ Average values of three biological repeats. In some cases, the SD could not be calculated because only 1 or none of the analyzed samples were positive for *E. amylovora* detection (NA, not applicable).

^e^ Quantity of target DNA copies per reaction well.

^f^ Total number of reaction wells in the chip that exceed the selected quality threshold.

^g^ Total number of positive calls for FAM dye in the chip, as determined from the Review Calls scatter plot. Based on the no-template control and plant material negative control analysis, chips with 5 or less than 5 positive calls were considered negative for *E. amylovora* detection, and the provided quantification values were not considered.

**Table S3.** Bacterial strains used in this study and specificity of the dPCR using different primer combinations.

|  |  | |  |  | **dPCR ^d^: Ams189R, Ams 141T and** | | | |
| --- | --- | --- | --- | --- | --- | --- | --- | --- |
| **Bacterial species and strains** | **Host plant** | **Geographic origin** | | **Isolator or reference** | **Ams119F** | **Ams03KbF** | **Ams06KbF** | **Ams09KbF** |
| ***Erwinia amylovora* strains ^a^** |  | |  |  |  |  |  |  |
| ATCC ^b^  49946 | *Malus pumila* | | USA | S. Beer (1971) | **+** | **+** | **+** | **+** |
| CFBP ^b^  1430 | *Crataegus* sp. | | France | Paulin & Samson (1973)^2^ | **+** | **+** | **+** | **+** |
| Ea 88-100 | *Pyrus communis* | | USA | Loper et al (1991)^3^ | **+** | **+** | **+** | **+** |
| E4001a | *M. pumila* | | USA | W. G. Bonn (1980) | **+** | **+** | **+** | **+** |
| NY17.1 | *M. pumila* | | USA | Russo et al (2008)^4^ | **+** | **+** | **+** | **+** |
| Ea DB | *M. pumila* | | USA | S.G. Aćimović (2016) | **+** | **+** | **+** | **+** |
| Ea DB8 | *M. pumila* | | USA | S.G. Aćimović (2016) | **+** | **+** | **+** | **+** |
| Ea ODFF7 | *M. pumila* | | USA | S.G. Aćimović (2016) | **+** | **+** | **+** | **+** |
| Ea ODFF14 | *M. pumila* | | USA | S.G. Aćimović (2016) | **+** | **+** | **+** | **+** |
| Ea CB15 | *M. pumila* | | USA | S.G. Aćimović (2016) | **+** | **+** | **+** | **+** |
| Ea RedCA13 | *Malus* spp. | | USA | S.G. Aćimović (2016) | **+** | **+** | **+** | **+** |
| Ea WhiteCA12 | *Malus* spp. | | USA | S.G. Aćimović (2016) | **+** | **+** | **+** | **+** |
| Ea CM9c1-B7 | *M. pumila* | | USA | R. Delgado Santander (2018) | **+** | **+** | **+** | **+** |
| Ea CM26c1-A1 | *M. pumila* | | USA | R. Delgado Santander (2018) | **+** | **+** | **+** | **+** |
| Ea CM26c1-D9 | *M. pumila* | | USA | R. Delgado Santander (2018) | **+** | **+** | **+** | **+** |
| Ea CM26c3-A1 | *M. pumila* | | USA | R. Delgado Santander (2018) | **+** | **+** | **+** | **+** |
| Ea F196r1 | *M. pumila* | | USA | Z. Pavlović (2018) | **+** | **+** | **+** | **+** |
| Ea RCM9r | *M. pumila* | | USA | R. Delgado Santander (2018) | **+** | **+** | **+** | **+** |
| Ea Ts1 | *M. pumila* | | USA | R. Delgado Santander (2018) | **+** | **+** | **+** | **+** |
| Ea RFs1 | *M. pumiila* | | USA | R. Delgado Santander (2018) | **+** | **+** | **+** | **+** |
| Ea SDs1 | *M. pumiila* | | USA | R. Delgado Santander (2018) | **+** | **+** | **+** | **+** |
| Ea SDs3 | *M. pumila* | | USA | R. Delgado Santander (2018) | **+** | **+** | **+** | **+** |
| Ea HCr1 | *M. pumila* | | USA | R. Delgado Santander (2018) | **+** | **+** | **+** | **+** |
| Ea HCc1 | *M. pumila* | | USA | R. Delgado Santander (2018) | **+** | **+** | **+** | **+** |
| Ea HCs1 | *M. pumila* | | USA | R. Delgado Santander (2018) | **+** | **+** | **+** | **+** |
| Ea JMs1 | *M. pumila* | | USA | R. Delgado Santander (2018) | **+** | **+** | **+** | **+** |
| Ea JMs3 | *M. pumila* | | USA | R. Delgado Santander (2018) | **+** | **+** | **+** | **+** |
| Ea RDs1 | *M. pumila* | | USA | R. Delgado Santander (2018) | **+** | **+** | **+** | **+** |
| Ea GGs1 | *M. pumila* | | USA | R. Delgado Santander (2018) | **+** | **+** | **+** | **+** |
| Ea HVRL^b^ s1 | *M. pumila* | | USA | R. Delgado Santander (2018) | **+** | **+** | **+** | **+** |
| **Other phytopathogeic bacteria** |  | |  |  |  |  |  |  |
| *Dickeya dadantii* |  | |  |  |  |  |  |  |
| 3937 | *Saintpaulia ionantha* | | France | M. Lemattre (1977) | **-** | **-** | **-** | **-** |
| *Dickeya dianthicola* |  | |  |  |  |  |  |  |
| ME23 | *Solanum tuberosum* | | USA | Ma et al (2007)^5^ | **-** | **-** | **-** | **-** |
| *Pectobacterium atrosepticum* |  | |  |  |  |  |  |  |
| SCRI1043 | *S. tuberosum* | | UK | De Boer & Sasser (1986)^6^ | **-** | **-** | **-** | **-** |
| *P. carotoborum* subsp. *carotoborum* |  | |  |  |  |  |  |  |
| WPP14 | *S. tuberosum* | | USA | Yap et al (2004)^7^ | **-** | **-** | **-** | **-** |
| *P. parmentiari* |  | |  |  |  |  |  |  |
| WPP163 | *S. tuberosum* | | USA | Ma et al (2007)^5^ | **-** | **-** | **-** | **-** |
| *Pantoea agglomerans* |  | |  |  |  |  |  |  |
| E325 | *M. pumila* | | USA | L. Pusey (1994) | **-** | **-** | **-** | **-** |
| Eh252 (CUCPB ^b^  2140) | *M. pumila* | | USA | Rundle & Beer (1987)^8^ | **-** | **-** | **-** | **-** |
| Eh318 (CUCPB ^b^  2050) | *M. pumila* | | USA | Wright et al. (2001)^9^ | **-** | **-** | **-** | **-** |
| *Pseudomonas syringae* pv. s*yringae* |  | |  |  |  |  |  |  |
| B728a | *Phaseolus vulgaris* | | USA | J. E. Loper & S. E. Lindow (1987) | **-** | **-** | **-** | **-** |
| HVRL ^b^  161 | *M. pumila* | | USA | Gašić et al (2018)^10^ | **-** | **-** | **-** | **-** |
| *P. syringae* pv*. tomato* |  | |  |  |  |  |  |  |
| DC3000 | *Lycopersicon esculentum* | | UK | Cuppels (1986)^11^ | **-** | **-** | **-** | **-** |
| **Saprophitic microbiota ^c^** |  | |  |  |  |  |  |  |
| Unknown bacterial species (5) | *M. pumila* | | USA | R. Delgado Santander (2018) | **-** | **-** | **-** | **-** |

^a^ Except the first five strains, all the *E. amylovora* isolates included in the primer specificity assays were isolated from 2016 to 2018 at the HVRL (Highland, NY, USA). All of them were pathogenic on apple leaves and pear slices, and positive for two species-specific PCR, one chromosomal^12^ and one detecting the almost ubiquitous plasmid pEA29^13^ .

^b^ ATCC, American Type Culture Collection; CFBP, Collection Française de Bactéries Phytopathogènes; CUCPB, Cornell University Collection of Phytopathogenic Bacteria; HVRL, Hudson Valley, Research Laboratory.

^c^ Saprophytic bacteria showing different colony morphology, and usually isolated together with *E. amylovora* from apple canker samples.

^d^ The primer probe concentrations and the thermal cycling in all cases, were the same as the ones described by Pirc et al.^1^. “+”, positive dPCR detection; “-”, negative dPCR detection.

**Table S4.** Apple and pear canker characteristics.

| **Host** | **Time period** | **Canker replicate** | **Branch diameter (cm)** | **Branch perimeter (cm)^a^** | **Canker length (cm)** | **Canker width (cm)^b^** | **Canker weight (g)^c^** | | **Volume of buffer (mL)^d^** | |
| --- | --- | --- | --- | --- | --- | --- | --- | --- | --- | --- |
| Apple cv. Honeycrisp | Mid-summer (2017) | 1 | 0.99 | 3.11 | 2.55 | G | 0.71 | 35.45 | |  |
|  |  | 2 | 2.70 | 8.48 | 5.36 | 2.80 | 0.24 | 12.05 | |  |
|  |  | 3 | 1.29 | 4.05 | 1.56 | G | 0.97 | 48.70 | |  |
|  | Mid-winter (2018) | 1 | 1.24 | 3.90 | 3.07 | 1.90 | 0.87 | 43.30 | |  |
|  |  | 2 | 2.21 | 6.94 | 5.68 | 4.00 | 2.69 | 134.35 | |  |
|  |  | 3 | 1.41 | 4.43 | 3.1 | 2.80 | 1.23 | 61.60 | |  |
| Pear cv. Bosc | Mid-summer (2017) | 1 | 0.63 | 1.98 | 1.31 | G | 0.36 | 17.85 | |  |
|  |  | 2 | 1.06 | 3.33 | 1.24 | 0.80 | 0.56 | 27.8 | |  |
|  |  | 3 | 1.96 | 6.16 | 2.19 | 1.40 | 1.47 | 73.6 | |  |
|  | Mid-winter (2018) | 1 | 0.59 | 1.85 | 0.49 | G | 0.19 | 9.65 | |  |
|  |  | 2 | 1.93 | 6.06 | 5.19 | G | 3.35 | 167.45 | |  |
|  |  | 3 | 1.47 | 4.62 | 4.1 | G | 1.54 | 77.1 | |  |

^a^ Branch circle perimeter, calculated as 2πr, where r is the branch radium at the measurement point.

^b^ G, the analyzed canker was girdling the branch, so its width equals to the branch perimeter.

^c^ Canker weight after slicing with a sterile scalpel, including the canker area plus a 4 mm wide ring outwards the canker margins.

^d^ The volume of buffer (0.1xAMB) was added in a ratio 1:50 (w/v) with respect to the canker weight.

**Table S5.** Checklist with the Minimum Information Required for the Minimum Information for Publication of Quantitative Digital PCR Experiments, according to Huggett et al.^14^.

| **ITEM TO CHECK** | **IMPORTANCE ^b^** | **Check** | **Comments** |
| --- | --- | --- | --- |
| **EXPERIMENTAL DESIGN** |  |  |  |
| Definition of experimental and control groups | **E** | **✓** | Materials and Methods |
| Number within each group | **E** | **✓** | Materials and Methods; |
| Assay carried out by core lab or investigator's lab? | D | **✓** | Investigator’s lab |
| Power analysis | D |  |  |
| Acknowledgement of authors' contributions | D |  |  |
| **SAMPLE** |  |  |  |
| Description | **E** | **✓** | Materials and Methods |
| Volume or mass of sample processed | **E** | **✓** | Materials and Methods |
| Microdissection or macrodissection | **E** | **✓** | Materials and methods |
| Processing procedure | **E** | **✓** | Materials and methods |
| If frozen - how and how quickly? | **E** | **✓** | Materials and methods |
| If fixed - with what, how quickly? | **E** | **✓** | N/A |
| Sample storage conditions and duration (especially for FFPE samples) | **E** | **✓** | Materials and methods |
| **NUCLEIC ACID EXTRACTION** |  |  |  |
| Procedure and/or instrumentation | **E** | **✓** | Materials and Methods |
| Name of kit and details of any modifications | **E** | **✓** | Materials and Methods |
| Manufacturer of reagents used and catalogue number | D | **✓** | Materials and Methods |
| Details of DNase or RNAse treatment | **E** | **✓** | The ones specified by the Dneasy Plant Mini Kit |
| Contamination assessment (DNA or RNA) | **E** | **✓** | Blank extraction controls (i.e. no sample input) were included in parallel with the samples to assess cross-contamination during extraction. |
| Nucleic acid quantification: Instrument and method | **E** | **✓** | When required, DNA was quantified with Qubit 2.0 BR dsDNA (Thermo Scientific) |
| Purity (A260/A280) | D | **✓** | N/A |
| Yield | D | **✓** | N/A |
| Electrophoresis traces | D | **✓** | N/A |
| Quality/integrity-instrument/method; e.g. RIN/RQI and trace or 3’:5’ | **E** | **✓** | N/A |
| Template structural information | **E** | **✓** | Pirc et al., 2009 |
| Template modification (digestion, sonication, pre-amplification etc.) | **E** | **✓** | N/A |
| Template treatment (initial heating or chemical denaturation) | **E** | **✓** | N/A |
| Inhibition dilution or spike; Cq dilutions | **E** | **✓** | N/A |
| Storage of nucleic acid: temperature, concentration, duration, buffer | **E** | **✓** | Nucleic acids were stored at -20°C in buffer AE (Qiagen) since DNA extraction, until use. |
| **REVERSE TRANSCRIPTION (If necessary)** |  |  |  |
| cDNA priming method/oligonucleotide + concentration | **E** | **✓** | N/A |
| One or two step protocol | **E** | **✓** | N/A |
| Reaction volume (for two step reverse transcription reaction) | D | **✓** | N/A |
| Detailed reaction components and conditions | **E** | **✓** | N/A |
| Amount of RNA used per reaction | **E** | **✓** | N/A |
| Reverse transcriptase and concentration | **E** | **✓** | N/A |
| Temperature and time | **E** | **✓** | N/A |
| RT efficiency | D |  |  |
| Estimated copies/Cq values measured with and without addition of RT* | D |  |  |
| Manufacturer of reagents used and catalogue number | D |  |  |
| Storage of cDNA: temperature, concentration, duration, buffer | D |  |  |
| **qPCR/dPCR TARGET INFORMATION** |  |  |  |
| Sequence accession number | **E** | **✓** | Pirc et al., 2009 |
| Location of amplicon | D | **✓** | Pirc et al., 2009 |
| Amplicon length | **E** | **✓** | Materials and Methods |
| In silico specificity screen (BLAST, etc) | **E** | **✓** | No off-target sequences identified. |
| Pseudogenes, retropseudogenes or other homologs? | D |  |  |
| Sequence alignment | D |  |  |
| Secondary structure analysis of amplicon and GC content | D |  |  |
| Location of each primer by exon or intron (if applicable) | **E** | **✓** | N/A |
| Where appropriate, which splice variants are targeted? | **E** | **✓** | N/A |
| qPCR: If multiplex, efficiency and LOD* of each assay. | **E** | **✓** | N/A |
| **qPCR/dPCR OLIGONUCLEOTIDES** |  |  |  |
| Primer sequences and/or amplicon context sequence** | **E** | **✓** | Materials and Methods |
| RTPrimerDB Identification Number | D |  |  |
| Probe sequences** | D | **✓** | Maerials and Methods |
| Location and identity of any modifications | **E** | **✓** | Pirc et al., 2009 |
| Manufacturer of oligonucleotides | D | **✓** | Invitrogen (Life technologies) |
| Purification method | D | **✓** | Desalted |
| **qPCR/dPCR PROTOCOL** |  |  |  |
| Complete reaction conditions | **E** | **✓** | Materials and Methods |
| Reaction volume and amount of RNA/cDNA/DNA | **E** | **✓** | Maerials and Methods |
| Primer, (probe), Mg++ and dNTP concentrations | **E** | **✓** | Maerials and Methods |
| Polymerase identity and concentration | **E** | **✓** | N/A (commercial mastermixes used) |
| Buffer/kit Catalogue No and manufacturer | **E** | **✓** | Maerials and Methods |
| Exact chemical constitution of the buffer | D |  |  |
| Additives (SYBR Green I, DMSO, etc.) | **E** | **✓** | N/A |
| Plates/tubes Catalogue No and manufacturer | D |  |  |
| Complete thermocycling parameters | **E** | **✓** | Maerials and Methods |
| Reaction setup (manual/robotic) | D | **✓** | Manual |
| Gravimetric or volumetric dilutions (manual/robotic) | D | **✓** | Volumetric |
| Master PCR reaction volume prepared | D | **✓** | Materials and Methods |
| Partition number | **E** | **✓** | QuantStudio 3D dPCR: up to 20,000 partitions |
| Individual partition volume | **E** | **✓** | 755 pL |
| Total volume of the partitions measured (effective reaction size) | **E** | **✓** | The number of partitions analyzed varied between each analyzed sample, and thus it needs to be determined individually with use of the information from QuantStudio 3D AnalysisSuite. In the present study, the number of accepted partitions ranged from 15,374 to 18,300, with a mean of 17,108. On the basis of their volume, the mean effective reaction size was estimated as 12.92 (±0.54) μL. |
| Partition volume variance/standard deviation | D |  |  |
| Comprehensive details and appropriate use of controls | **E** | **✓** | Materials and Methods |
| Manufacturer of dPCR instrument | **E** | **✓** | Materials and Methods |
| **qPCR/dPCR VALIDATION** |  |  |  |
| Optimisation data for the assay | D |  |  |
| Specificity (when measuring rare mutations, pathogen sequences etc.) Gel, sequence, melt, or digest | **E** | **✓** | Specificity determined *in silico* using NCBI BLAST and *in vitro* (Materials and Methods; Supplementary Table S3) |
| If multiplexing, comparison with singleplex assays | **E** | **✓** | N/A |
| qPCR SYBR Green I: Cq of the NTC | **E** | **✓** | N/A |
| qPCR standard curves with slope and y-intercept | **E** | **✓** | N/A |
| PCR efficiency calculated from slope | **E** | **✓** | N/A |
| Confidence interval for PCR efficiency or standard error | D |  |  |
| r2 of standard curve | **E** | **✓** | N/A |
| qPCR linear dynamic range | **E** | **✓** | N/A |
| Cq variation at lower limit | **E** | **✓** | N/A |
| Confidence intervals throughout range | D |  |  |
| dPCR: Evidence for limit of detection | **E** | **✓** | Results section |
| Limit of detection of dPCR calibration control | D | **✓** | Results section |
| **DATA ANALYSIS** |  |  |  |
| Average copies per partition (λ or equivalent ) | **E** | **✓** | Results section |
| Cq method determination | **E** | **✓** | N/A |
| qPCR/dPCR analysis program (source, version) | **E** | **✓** | Materials and methods |
| Outlier identification and disposition | **E** | **✓** | Materials and methods |
| Results of NTCs | **E** | **✓** | Available upon request |
| Examples of positive(s) and negative experimental results as supplemental data | **E** | **✓** | Supplementary Figs. S1, S2 |
| Where appropriate, justification of number and choice of reference genes | **E** | **✓** | N/A |
| Where appropriate, description of normalisation method | **E** | **✓** | Materials and Methods |
| Number and concordance of biological replicates | D | **✓** | Materials and Methods |
| Number and stage (RT or qPCR) of technical replicates | **E** | **✓** | Materials and Methods |
| Repeatability (intra-assay variation) | **E** | **✓** | Results section |
| Reproducibility (inter-assay/user/lab etc. variation ) | D | **✓** | N/A |
| Experimental variance or confidence interval | **E** | **✓** | Results section |
| Statistical methods used for analysis | **E** | **✓** | Materials and Methods |
| Data submission using RDML | D |  |  |

^a^ E, essential information; D, desirable information

**Supplementary Figures**

**Figure S1. QS3D dPCR 2D scatter plots representative of a negative and a positive control.** No-template negative control prepared with the reaction mix plus water (a). Positive control containing *E. amylovora* ATCC 49946 DNA extracted from an overnight culture in LB. Fluorescence values of the FAM labelled probe in wells with positive dPCR reactions are blue, while wells with negative amplifications are yellow.

**Fig. S2. Representative dPCR 2D scatter plots corresponding to plant material negative and positive controls.** For negative control preparation (a,b,e,f), *E. amylovora-*free apple (a-d) and pear (e-h) macerates were prepared in AMB. Positive controls were inoculated with 10^6^ CFU mL^-1^ of *E. amylovora* ATCC 49946 (left) or CFBP 1430 (right), and subjected to DNA extraction and dPCR, using the same primer/probe concentrations and thermal cycling conditions as a previous work on qPCR^1^. Fluorescence values of the FAM labelled probe in wells with positive dPCR amplifications are blue, while wells with negative amplifications are yellow.


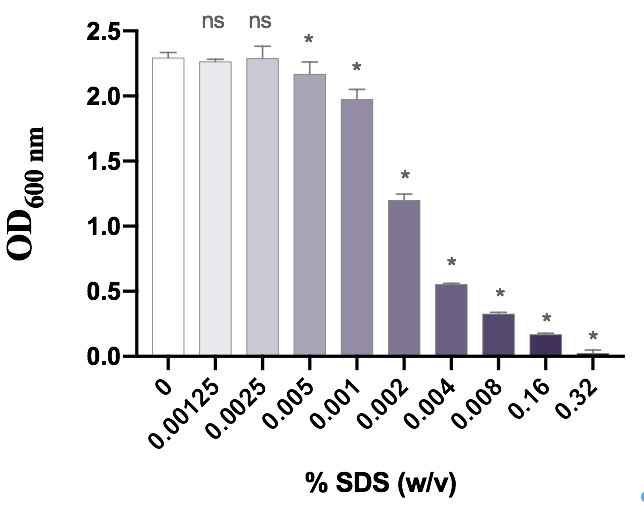


**Fig. S3. SDS MIC assay on *E. amylovora* ATCC 49946.** The assay was performed in fresh LB amended with two-fold increasing concentrations of SDS, from 0 to 0.32 % (w/v). The medium was inoculated with a 1/100 dilution of an *E. amylovora* overnight culture in LB, and incubated at 28 ºC with shaking (180 rpm) for 16 h. Afterwards, the effect of SDS on *E. amylovora* growth was measured by means of reduction in the OD_600_ nm with respect to a control lacking SDS. Each column shows the mean values of an experiment performed in quadruplicate, and error bars denote the SD. Statistically significant differences between the OD_600_ nm values of the control and each SDS concentration were calculated by a non-parametric Mann-Whitney test (unpaired, two-tailed), and are indicated above the columns with asterisks (*, p < 0.05); ns, non-significant differences.


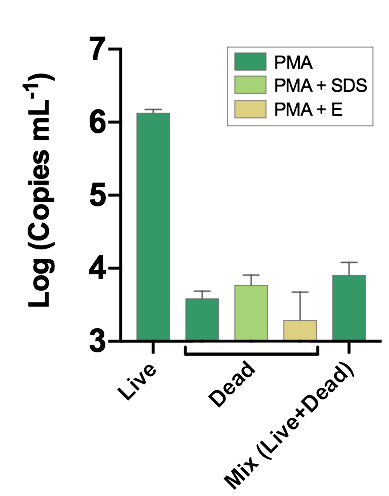


**Fig. S4. False positive detection by v-dPCR in samples composed of dead *E. amylovora* cells using the primer pair Ams03KbF/Ams189R.** Apple branch (cv. Honeycrisp) macerates were prepared with 0.1xAMB, and inoculated either with *E. amylovora* (ATCC 49946) live cells (10^6^ CFU mL^-1^), dead cells (10^6^ dead cell mL^-1^) or a mixture of live (10^3^ CFU mL^-1^) and dead cells (10^6^ dead cell mL^-1^). Samples were treated either with PMA, PMA plus SDS (PMA+SDS), or PMA plus PMA Enhancer for Gram Negative Bacteria (PMA+E). Afterwards, DNA was extracted, and v-dPCR performed using the primer pair Ams03KbF/Ams189R and the probe Ams141T. Results are average values of three biological repeats of the same experiment.


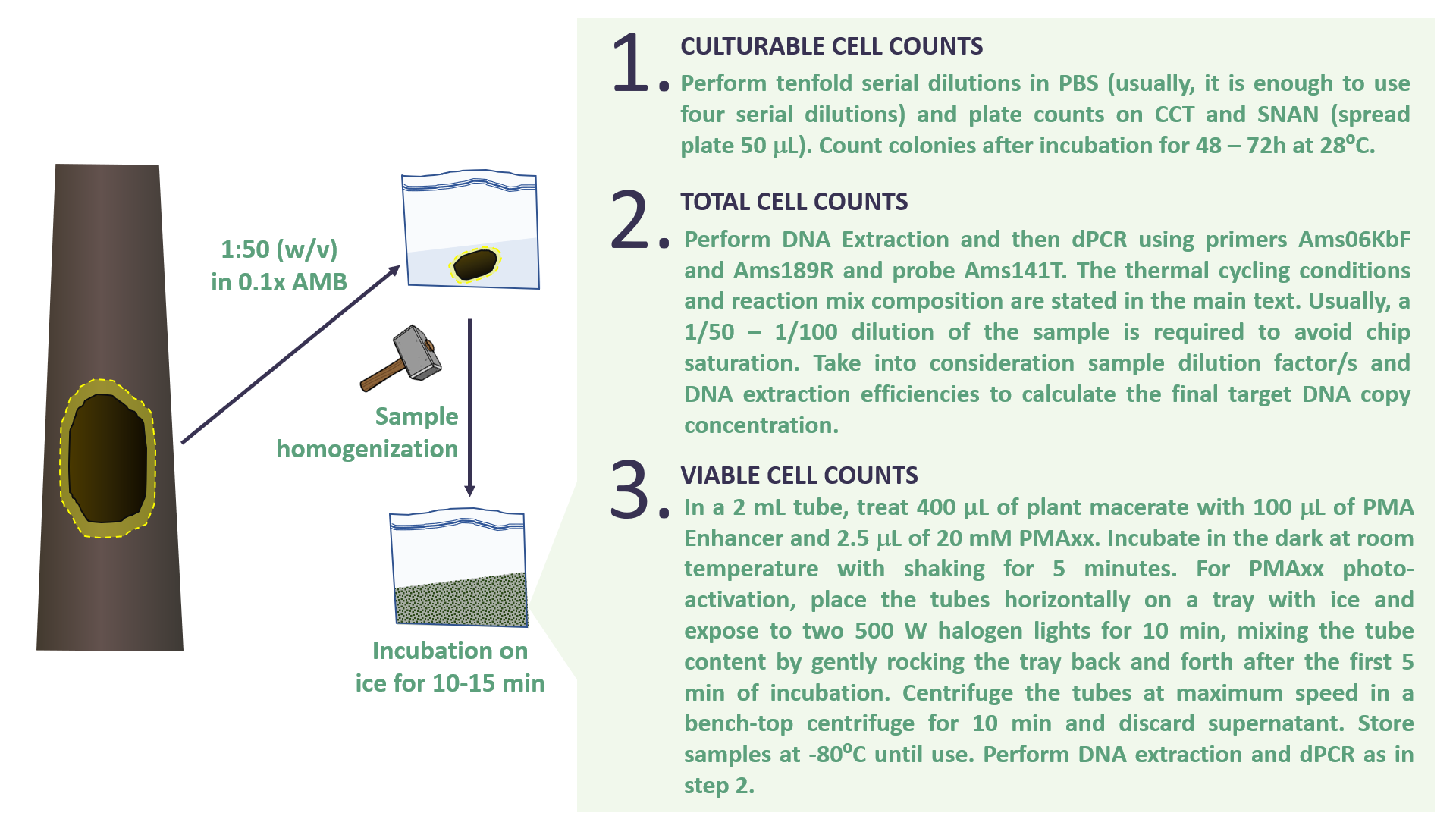


**Fig. S5. Final protocol for sample processing and *E. amylovora* total, live and culturable cell population analysis in natural cankers.**

**References**

1. Pirc, M., Ravnikar, M., Tomlinson, J. & Dreo, T. Improved fireblight diagnostics using quantitative real-time PCR detection of *Erwinia amylovora* chromosomal DNA. *Plant Pathol.* **58,** 872–881 (2009).

2. Paulin, J. P. & Samson, R. Le feu bactérien en france II. Caractères des souches d'*Erwinia amylovora* (Burril) Winslow *et al.*, 1920, isolées du foyer franco-belge. *Ann. Phytopathol.* **5,** 389–397

3. Loper, J. E., *et al.* Evaluation of streptomycin, oxytetracycline, and copper resistance of *Erwinia amylovora* isolated from pear orchards in Washington State. *Plant Dis*. **75,** 287-290 (1991).

4. Russo, N. L., Burr, T. J., Breth, D. I. & Aldwinckle, H. S. isolation of streptomycin-resistant isolates of *Erwinia amylovora* in New York. *Plant Dis.* **92,** 714–718 (2008).

5. Ma, B., *et al*. Host range and molecular phylogenies of the soft rot enterobacterial genera *Pectobacterium* and *Dickeya*. *Phytopathology*, **97,** 1150–1163 (2007).

6. De Boer, S. H., & Sasser, M. Differentiation of *Erwinia carotovora* subsp. *carotovora* and *E. carotovora* subsp. *atroseptica* on the basis of cellular fatty acid composition. *Can. J. Microbiol.* **32,** 796–800 (1986).

7. Yap, M.-N., Barak, J. D. & Charkowski, A. O. Genomic diversity of *Erwinia carotovora* subsp. *carotovora* and Its correlation with virulence. *Appl. Environ. Microbiol.* **70,** 3013–3023 (2004).

8. Rundle, J. R. & Beer, S. V. Population dynamics of *Erwinia amylovora* and a biological control agent, *Erwinia herbicola*, on apple blossom parts. *Acta Hortic.* **217,** 221–222 (1987).

9. Wright, S. A. I., Zumoff, C. H., Schneider, L. & Beer, S. V. *Pantoea agglomerans* strain eh318 produces two antibiotics that inhibit *Erwinia amylovora* in vitro. *Appl. Environ. Microbiol.* **67,** 284–292 (2001).

10. Gašić, K., Pavlović, Ž., Santander, R., Meredith, C. & Aćimović, S. G. First report of *Pseudomonas syringae* pv. *syringae* associated with bacterial blossom blast on apple (*Malus pumila*) in USA. *Plant Dis.* **102,** 1848-1848 (2018).

11. Cuppels, D. A. Generation and Characterization of Tn5 Insertion Mutations. *Appl. Environ. Microbiol.* **51,** 323–327 (1986).

12. Taylor, R., Guilford, P., Clark, R., Hale, N. & Forster, R. Detection of *Erwinia amylovora* in plant material using novel polymerase chain reaction (PCR) primers. *New Zeal. J. Crop Hortic. Sci.* **29,** 35–43 (2001).

13. Bereswill, S., Pahl, A., Bellemann, P., Zeller, W. & Geider, K. Sensitive and species-specific detection of Erwinia amylovora by polymerase chain reaction analysis. *Appl. Environ. Microbiol.* **58,** 3522–3526 (1992).

14. Huggett, J. F. *et al.* The Digital MIQE Guidelines: Minimum Information for Publication of Quantitative Digital PCR Experiments. *Clin. Chem.* **59,** 892–902 (2013).
